# Video game rehabilitation for outpatient stroke (VIGoROUS): A multi-site randomized controlled trial of in-home, self-managed, upper-extremity therapy

**DOI:** 10.1016/j.eclinm.2021.101239

**Published:** 2021-12-17

**Authors:** Lynne V. Gauthier, Deborah S. Nichols-Larsen, Gitendra Uswatte, Nancy Strahl, Marie Simeo, Rachel Proffitt, Kristina Kelly, Roger Crawfis, Edward Taub, David Morris, Linda Pax Lowes, Victor Mark, Alexandra Borstad

**Affiliations:** a University of Massachusetts Lowell, Dept. Physical Therapy and Kinesiology; b The Ohio State University, School of Health and Rehabilitation Sciences; c University of Alabama Birmingham, Dept. of Psychology; d Providence Medford Medical Center; e OhioHealth Neurological Rehabilitation; f University of Missouri, Dept. of Occupational Therapy; g Ohio State University, Dept of Neurology; h Ohio State University, Dept of Computer Science; i University of Alabama Birmingham, Dept. of Physical Therapy; j Nationwide Children's Hospital; k University of Alabama Birmingham, Dept. of Physical Medicine and Rehabilitation; l College of St. Scholastica, Dept. of Physical Therapy

## Abstract

**Background:**

Integrating behavioral intervention into motor rehabilitation is essential for improving paretic arm use in daily life. Demands on therapist time limit adoption of behavioral programs like Constraint-Induced Movement (CI) therapy, however. Self-managed motor practice could free therapist time for behavioral intervention, but there remains insufficient evidence of efficacy for a self-management approach.

**Methods:**

This completed, parallel, five-site, pragmatic, single-blind trial established the comparative effectiveness of using in-home gaming self-management as a vehicle to redirect valuable therapist time towards behavioral intervention. Community-dwelling adults with post-stroke (>6 months) mild/moderate upper extremity hemiparesis were randomized to receive one of 4 different interventions over a 3-week period: 5 h of behaviorally-focused intervention plus gaming self-management (Self-Gaming), the same with additional behaviorally-focused telerehabilitation (Tele-Gaming), 5 h of Traditional motor-focused rehabilitation, or 35 h of CI therapy. Primary outcomes assessed everyday arm use (Motor Activity Log Quality of Movement, MAL) and motor speed/function (Wolf Motor Function Test, WMFT) immediately before treatment, immediately after treatment, and 6 months later. Intent-to-treat analyses were implemented with linear mixed-effects models on data gathered from March 15, 2016 to November 21, 2019. ClinicalTrials.gov, NCT02631850.

**Results:**

Of 193 enrolled participants, 167 began treatment and were analyzed, 150 (90%) completed treatment, and 115 (69%) completed follow-up. Tele-Gaming and Self-Gaming produced clinically meaningful MAL gains that were 1·0 points (95% CI 0·8 to 1·3) and 0·8 points (95% CI 0·5 to 1·0) larger than Traditional care, respectively. Self-Gaming was less effective than CI therapy (-0·4 points, 95% CI -0·6 to -0·2), whereas Tele-Gaming was not (-0·2 points, 95% CI -0·4 to 0·1). Six-month retention of MAL gains across all groups was 57%. All had similar clinically-meaningful WMFT gains; six-month retention of WMFT gains was 92%.

**Interpretation:**

Self-managed motor-gaming with behavioral telehealth visits has outcomes similar to in-clinic CI therapy. It addresses most access barriers, requiring just one-fifth as much therapist time that is redirected towards behavioral interventions that enhance the paretic arm's involvement in daily life.

**Funding:**

10.13039/100006093PCORI, NIH

**Research in context**

**Evidence before this study**

A systematic review of 14 meta-analyses and randomized controlled trials (RCTs) in PubMed, Cochrane Library, Medline, and Ovid revealed Level 1a evidence that incorporating behavioral intervention within dose-matched neurologic upper-extremity motor rehabilitation produces clinically-meaningful improvement in activities of daily living (search terms: “stroke”; “hemiplegi*” or “upper limb” or “upper extremity” or “arm” or “hand” or “motor” or “hemiparesis” or “paretic”; “therapy” or “intervention” or “rehabilitation”; and “behavior*” or "transfer package" or "problem-solving" or "problem solving" published up to June 12, 2021). We searched the same databases to determine the effectiveness of self-administering upper-extremity practice (search terms: “stroke”; and “self-manage*” or "self manage*" or “non-professional” or "independent practice" or “self-guided” or "self guided”; and “motor” or “hemiparesis” or “arm” or “hand“ or "upper extremity” or “paretic”; and “rehabilitation” or “intervention” or “therapy” or “treatment” or “intervention”; and “home” or “outpatient” or “community” or “out-patient”). Two trials compared self-managed versus therapist-led motor practice, with neither showing meaningful differences in function or use compared to either lower-dose traditional care or dose-matched CI therapy.

**Added value of this study**

Incorporating behavioral intervention into post-stroke upper-extremity rehabilitation is critical, but evidence-based approaches are not clinically feasible. We addressed this gap by allocating therapist time entirely to behavioral intervention, while motor practice was self-managed at home through video games. We present here a multi-site pragmatic randomized controlled trial of such a “flipped” model of care for chronic upper-extremity paresis. Arm use for daily activities improved to a similar extent as time-intensive in-clinic CI therapy and more than traditional motor-focused care; motor gains were similar.

**Implications of all the available evidence**

Allocating scarce therapy time to behavioral intervention yields superior improvements in arm use. Self-management through rehabilitation gaming improves motor function to the same extent as in-clinic motor practice.

Alt-text: Unlabelled box

## Introduction

The majority of stroke survivors experience prolonged difficulty using the paretic arm and hand to complete daily activities.^1^ Motor training typically reduces impairment, but does not restore habitual use of the paretic arm,^2^,^3^ limiting its impact on a person's ability to complete everyday activities. Adding specific behavioral interventions (self-monitoring, contracting, goal-setting, and problem-solving, eTable 1) to a dose-matched rehabilitation program promotes greater involvement of the paretic arm in daily activities.^2^ Comprehensive interventions that incorporate both behavioral and motor interventions, such as Constraint-Induced Movement Therapy (CI therapy), accordingly produce much larger gains in everyday arm use than interventions that only emphasize intensive motor practice.^2^,^4^,^5^

Many stroke survivors cannot access intensive and comprehensive behavioral/motor interventions, however, and thus fail to progress, or even functionally decline, after inpatient discharge.^6^ Major barriers include geographic location (e.g., rural disparities), travel-burden, insurance restrictions, and the time cost of delivering both behavioral and motor intervention.^6^,^7^ The reduction of legal/regulatory barriers to telerehabilitation following COVID-19 poses a unique opportunity to explore accessible and efficient models of care that can overcome these challenges.

The expansion of telerehabilitation also provides an opportunity to reframe the role of Physical and Occupational therapists in upper limb rehabilitation. The prevailing model of care emphasizes motor practice, with minimal emphasis on methods to induce behavior change. Early evidence indicates that motor practice can be self-managed,^8^, ^9^, ^10^ while successful behavioral change requires extensive therapist support.^2^,^4^ Taken together, this prior work suggests that behavioral intervention may be the most impactful and efficient use of therapist time.

Accordingly, our team leveraged new gaming technology and telerehabilitation to flip the balance of therapist time spent on motor practice versus behavioral intervention. In this new model of care, therapist time is primarily dedicated to behavioral interventions targeting arm use, which can be carried out through telerehabilitation to improve access. Self-managed intensive motor practice occurs in-home through rehabilitation gaming technology that progresses difficulty, provides immediate feedback, and tracks progress.^11^ This flipped model of care proved safe,^9^,^12^ feasible, and was preferred by clients.^9^ A definitive pragmatic randomized controlled trial was still needed, however, to determine whether devoting therapist time almost entirely to behavioral intervention could be as effective as traditional rehabilitation that primarily utilizes therapist time for motor practice. Moreover, the efficacy of this self-managed, time-efficient approach needed to be contrasted with a comprehensive time-intensive intervention (CI therapy) in which a therapist delivers both motor practice and behavioral treatment.

The primary objective of this pragmatic multi-site randomized 4 parallel-arm trial was to determine the effect that leveraging telerehabilitation technology to allocate therapist time differently has on the function and use of the more-impaired upper extremity. Two in-home self-managed video game-based treatments (in which therapists deliver behavioral treatment) are compared to traditional therapist-supervised rehabilitation (where therapists deliver motor practice) and to CI therapy (in which therapists deliver both behavioral treatment and motor practice). The hypothesis was that this new, flipped model of behavior-centric self-managed game-based therapy would improve function (WMFT) and daily activities performance (MAL) of the paretic upper extremity to the same extent as in-clinic CI therapy and more than Traditional motor-centric rehabilitation.

## Methods

### Study design

This parallel, four-arm, five-site, pragmatic, single-blind trial with 6-month follow-up was carried out within sociogeographically-diverse outpatient neurorehabilitation settings at three academic medical centers and two community rehabilitation clinics throughout the USA. The Internal Review Boards at The Ohio State University, Missouri University, Providence Medford Medical Center, University of Alabama Birmingham, and OhioHealth provided ethical oversight for the research (Research Protocol).

### Participants

Community dwelling stroke survivors experiencing chronic mild to moderate upper extremity hemiparesis were recruited from Feb 2016 through May 2019. Active range of motion criteria included > 10° in at least 2 fingers, thumb, and wrist; > 45° shoulder abduction and flexion; > 20° elbow extension. Eligible participants were adults who had experienced a stroke of any etiology at least 6 months prior to enrollment, were able to provide informed written consent, and were able/willing to commit to whichever three-week treatment protocol they were randomized to (eTable 2).

### Randomization and masking

Participants were initially stratified by baseline motor ability (whether or not they could place any number of pegs on the 9-Hole Peg Test within 120 s) prior to randomization. The randomization procedure involved the participant drawing a sealed paper containing their group assignment from a large opaque envelope positioned overhead by a study coordinator (Supplement-4.2).^13^ Participants were masked to the study hypothesis. Assessors were masked to group assignment. Participants were instructed not to disclose any details of their treatment to the assessor. Fidelity of masking was assessed through tape-recordings of assessment sessions.

### Interventions

The four-group pragmatic trial design aimed to determine the comparative effectiveness of a more accessible form of CI therapy (involving self-managed video-game motor practice at home and intermittent behaviorally-focused in-clinic treatment) relative to its resource-intensive CI therapy predecessor^2^ and Traditional motor-focused care (Table 1). Two in-home gaming groups received behavioral intervention targeting everyday use of the more impaired arm during in-clinic visits of similar frequency/duration to traditional rehabilitation;^14^ motor practice was entirely self-managed at home through a video game. One of the in-home gaming groups received additional therapist contact via telehealth (Tele-Gaming group). A Traditional rehabilitation active comparator group received the same schedule of in-clinic treatment as the gaming groups, but treatment was focused instead on traditional motor interventions. The comparison between the gaming groups and the Traditional group thus examined the impact of leveraging self-management with gaming technology to allocate therapist time for behavioral intervention. A CI therapy active comparator group received more frequent and intensive intervention in which the therapist delivered both behavioral and motor interventions in the clinic. The comparison between the gaming groups and the CI therapy group thus assessed whether the motor practice portion of CI therapy could be as effectively self-managed at home.

**Table 1:** Treatment group comparison. Treatments occurred over a 3-week period. *h* = hours.

	Self-Gaming	Tele-Gaming	CI therapy	Traditional
In-clinic intervention	5 h, behavioral focus	5 h, behavioral focus	35 h behavioral & motor focus	5 h, motor focus
Prescribed structured motor intervention	15 h (game)	15 h (game)	15 h (in-clinic)	5 h (in-clinic)
Therapist consults	4	10	10	4
Time in behavioral intervention	5 h	7.6 h	5–7 h	0 h
Rest	0 h	0 h	13–15 h	Only as needed
Total therapist time	5 h	7•6 h (5 h in-clinic plus 2•6 h for the 6 tele-health consults)	35 h	5 h
Prescribed home practice	5 h task practice	5 h task practice	5 h task practice	5 h strengthening

**Table 2:** Baseline characteristics of the intent-to-treat sample.

Demographic and Clinical Characteristics (*N* = 167)	Self-Gaming	Tele-Gaming	CI therapy	Traditional
	(*n* = 44)	(*n* = 45)	(*n* = 40)	(*n* = 38)
**Male**	24 (55%)	26 (58%)	30 (75%)	30 (79%)
**Right Hand Affected**	26 (59%)	15 (33%)	24 (60%)	21 (55%)
**Rural** (*n* = 164)	11 (25%)	16 (37%)	10 (26%)	15 (39%)
**Caucasian** (*n* = 160)	27 (63%)	30 (67%)	29 (72%)	24 (63%)
**African American** (*n* = 160)	14 (33%)	9 (20%)	9 (22%)	10 (26%)
**Asian** (*n* = 160)	1 (2%)	4 (9%)	0 (0%)	2 (5%)
**Other race/ethnicity** (*n* = 160)	0 (0%)	0 (0%)	1 (2%)	0 (0%)
**Diminished light touch**	17 (39%)	12 (27%)	17 (42%)	16 (42%)
**Diminished protective sensation**	5 (11%)	10 (22%)	8 (20%)	2 (5%)
**No protective sensation**	8 (18%)	9 (20%)	10 (25%)	15 (39%)
**Mild cognitive impairment**	19 (43%)	13 (30%)	17 (42%)	19 (50%)
**Very poor cognition**	3 (7%)	6 (14%)	6 (15%)	7 (18%)
**MoCA total** (*n* = 166)	22·3 (5·4)	22·5 (5·6)	21·6 (6·4)	20·1 (6·0)
**Age (years)**	60 (14)	56 (17)	62 (13)	63 (14)
**Chronicity (years)**	5·2 (6·5)	3·4 (5·1)	4·9 (9·8)	5·8 (8·1)
**Baseline WMFT**	1·6 (1·0)	1·6 (0·9)	1·8 (1·1)	1·8 (0·9)
**Baseline MAL**	1·5 (0·8)	1·5 (0·9)	1·5 (1·0)	1·2 (0·8)

Data are n (%), mean (SD).

Rural = address designated rural via Centers for Medicare & Medicaid Services Rural Health Clinic criteria.

Diminished light touch = detected 0·16 – 0·4 g of pressure on the Semmes-Weinstein Monofilament test.

Diminished protective sensation = detected 0·6 – 2 g of pressure on the Semmes-Weinstein Monofilament test.

No protective sensation = cannot detect < 4 g of pressure on the Semmes-Weinstein Monofilament test.

MoCA = Montreal Cognitive Assessment total score.

Mild cognitive impairment = score of less than 24 on the MoCA.

Very poor cognition = score of less than 16 on the MoCA.

Chronicity = Years between stroke and study participation.

WMFT = Wolf Motor Function Test mean natural log transformed performance time.

MAL = mean Motor Activity Log score.

*Tele-Gaming and Self-Gaming:* The two gaming groups received 4 visits (5 h) over 3 weeks of in-clinic one-on-one treatment with a therapist. This schedule of in-clinic therapist contact simulates the scarce access to therapist intervention that most people with chronic stroke experience, ^14^ particularly those who are underinsured, reside in rural areas, or lack transportation. Therapy visits had an almost exclusive emphasis on behavioral intervention. Behavioral interventions included identifying treatment goals that are personally meaningful, motivational interviewing to reinforce commitment to habit change, signing a treatment contract committing to involve the paretic arm in the majority of daily tasks, breaking down activities of daily living into detailed component tasks, listing these component tasks along with a plan for how the paretic arm will be used for each, recording use of the paretic arm for each of the listed component tasks between therapy visits (self-monitoring), reviewing this list during each treatment session to promote accountability, additional self-monitoring/feedback via informal administration of the Motor Activity Log, and guided problem-solving to overcome barriers to using the paretic arm (eTable 1).^2^ To further develop capacity to perform specific tasks related to their treatment goals, participants independently practiced goal-directed tasks for 30 min on 10 separate days between therapy visits.^2^ In lieu of the CI therapy restraint mitt, a smart watch worn on the paretic arm provided vibration feedback and a “please use me” notification when more than 10 min of inactivity was detected.

The Self-Gaming group received no therapist contact between clinic visits. The Tele-Gaming group received 6 additional brief behavioral video-consultations, totaling 2·6 h, between clinic visits. Video-consultations focused primarily on problem-solving around barriers to using the paretic arm during daily life (Table 1). The Tele-Gaming group thus received the same frequency of therapist contact supporting behavioral change as the CI therapy group, while the Self-Gaming group received the same frequency of therapist contact as the Traditional group.

New low-cost commercially available (Games That Move You, PBC) interactive video gaming technology provided engaging self-managed home practice (eFig. 1).^9^ Both gaming groups were provided with a gaming system at the initial visit. They were prescribed 15 h of unsupervised game play driven by movements of the paretic arm, the same dose of active motor practice administered to the CI therapy comparator. Consistent with the motor learning principles employed by CI therapy,^4^,^10^ the game provided frequent feedback and progressed task difficulty automatically based on performance (eTables 4 & 5). All participants received a balanced upper extremity program consisting of all of the following in-game movements, executed both separately and in combination: shoulder flexion/extension, shoulder abduction, horizontal shoulder adduction across midline, elbow flexion/extension, forearm supination, grasp/release, finger flexion/extension and thumb abduction/adduction, wrist extension, and targeted reaching (eTable 4). Therapists could customize the relative balance of shoulder, elbow, wrist, and hand movements through the game's user interface to suit the needs/goals of each participant.

**Fig. 1:**
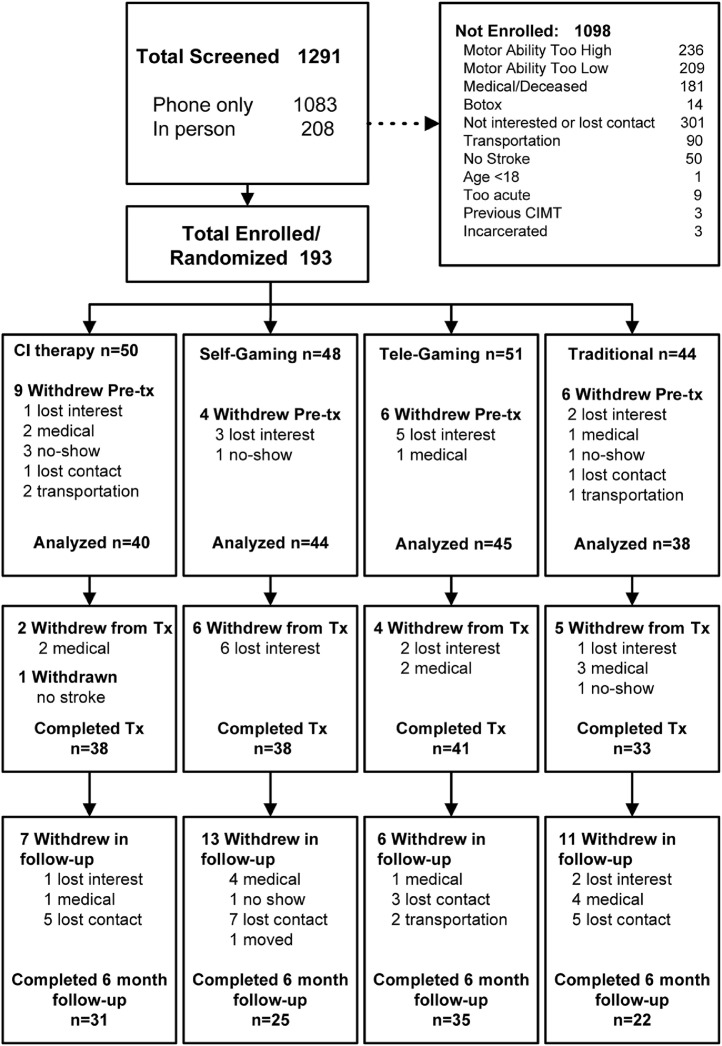
CONSORT flow diagram detailing recruitment, reasons for exclusion, and breakdown of attrition.

*Traditional therapist-supervised rehabilitation (Traditional)* involved the same frequency and duration of in-clinic treatment as the two gaming groups (5 h, 4 visits), with a traditional focus on motor training. Sessions involved neuromuscular reeducation, functional training, progressive strengthening, teaching a home program, and rest as needed to maintain a target for exercise intensity of 4 (somewhat hard) on the Borg CR10 Rating of Perceived Exertion Scale. A self-managed home program consisted of 15 min of strengthening exercises twice daily on the first 10 non-treatment days, to mirror the intensity and type of home practice that is routinely prescribed in standard clinical practice. The duration of home practice for the Traditional group also matched the time that the gaming groups spent on behavioral home practice. Visual aids (e.g., booklet of printed exercises) and typical therapy supplies (e.g., Theraband) were provided to participants to assist them in carrying out their home programs.

After the 6-month follow-up assessment, this group was offered access to 3 weeks of gaming self-management. The rationale for including this cross-over treatment was to reduce attrition by offering a novel treatment to participants in this group and to determine the feasibility of self-managing both the motor and behavioral components of the intervention. Participants received a single two-hour therapist consultation training them to use the gaming system and were introduced to the behavioral interventions (goal setting, contract, problem-solving, behavioral home practice, eTable 1). They received a 30-minute video outlining the importance of the behavioral interventions and how to implement them, a computerized application to facilitate self-monitoring arm use, and the same forms (e.g., for self-monitoring and problem-solving) that were employed for the Self-Gaming and Tele-Gaming interventions. They then had to self-manage both gaming practice (target 15 h) and the behavioral interventions.

*CI therapy* is an established treatment that formed the foundation of evidence for the importance of behavioral intervention within motor rehabilitation.^2^,^4^ This comparator reflects the improvement that can be achieved from behavioral/motor intervention under ideal conditions in which therapist time, cost, and access are not constrained. CI therapy involved ten 3·5 hour in-clinic treatment sessions over three weeks. Each session included 1·5 h of *active* motor practice with progression of task difficulty and continuous performance feedback (15 h total, consistent with the gaming prescription). Rest was interspersed throughout the motor practice portion of the session (e.g., between repetitions, during task-set-up) such that there was an approximate 50:50 balance between time spent in active motor practice versus rest. The CI therapy group received the same package of behavioral interventions as the gaming groups (during each of 10 sessions). Time spent in behavioral intervention was not rigidly constrained (there was greater emphasis on administering the required elements than on time spent), but total time spent on behavioral intervention typically ranged from 5–7 h, consistent with the gaming groups. Participants were prescribed a padded mitt restraint to wear on the less affected hand for 10 h daily. Behavioral home practice matched that of the gaming groups.

All treatments were supervised by licensed physical or occupational therapists. Therapist training, supervision, periodic review of video-taped sessions, periodic re-training, and checklists promoted adherence to the Research Protocol. See Table 1, Supplement 4.3–4.4, eTable 3 for detailed descriptions of the treatments and fidelity checks.

**Table 3:** Means and SDs of each treatment group at each time-point and the comparative treatment effects.

Mean (standard deviation) of each treatment group at each time-point					
*N* = 167		Self-Gaming (*n* = 44)	Tele-Gaming (*n* = 45)	CI therapy (*n* = 40)	Traditional (*n* = 38)
MAL	Pre	1·5 (0·8)	1·5 (0·9)	1·5 (1)	1·2 (0·8)
	Post	2·8 (0·9)	3·1 (0·9)	3·2 (1)	1·7 (1·1)
	Follow-up	2·1 (1·0)	2·4 (1·2)	2·6 (1·2)	1·6 (1·2)
WMFT a	Pre	1·64 (1·05)	1·60 (0·91)	1·82 (1·07)	1·81 (0·92)
	Post	1·40 (1·03)	1·31 (0·75)	1·44 (0·97)	1·60 (0·95)
	follow-up	1·43 (0·97)	1·35 (0·78)	1·50 (0·98)	1·54 (0·91)
**Comparative Treatment Effects (95% CI)**^b^					
		**Self-Gaming vs CI therapy**	**Self-Gaming vs Traditional**	**Tele-Gaming vs CI therapy**	**Tele-Gaming vs Traditional**
MAL Post		−0·4 s (−0·6, −0·2)	0·8 s (0·5, 1·0)	−0·2 (−0·4, 0·1)	1·0 s (0·8, 1·3)
MAL Follow-up		−0·5 s (−0·8, −0·2)	0·3 (−0·0, 0·7)	−0·2 (−0·6, 0·1)	0·6 s (0·2, 1·0)
WMFT Post		0·18 (−0·00, 0·37) 0·85 s/task	0·01 (−0·18, 0·20) 0·10 s/task	0·13 (−0·05, 0·32) 0·71 s/task	−0·04 (−0·22, 0·15) −0·03 s/task
WMFT Follow-up		0·21 (−0·15, 0·57) 0·71 s/task	0·15 (−0·21, 0·52) 0·47 s/task	0·19 (−0·17, 0·55) 0·59 s/task	0·14 (−0·23, 0·50) 0·35 s/task

a mean of the natural log of performance times for each item (SD).

b Estimated between-group differences in change from baseline for each outcome, adjusted for covariates in the final mixed effects general linear model (95% confidence interval). A positive between-group difference for the MAL means that the gaming group reported greater gains in quality of arm use than the comparison group. A positive between-group difference for the WMFT means that the gaming group showed worse improvements in performance time than the comparison group. WMFT between-group effects are additionally converted to differences in mean performance time per task to ease clinical interpretation.

s Statistically significant differences between-groups.

### Assessments

*Timeline:* Participants were assessed within one week prior to and following treatment, and again 5–7 months post-treatment. The Traditional group was additionally reassessed at 6–8 months post-treatment after completing the gaming cross-over to support exploratory analyses of a fully self-managed gaming protocol. Examiners were naïve to group assignment.

*Arm Use (Primary outcome):* The Motor Activity Log Quality of Movement scale (MAL) is a reliable and valid structured interview of quality of arm use for 28 activities of daily living.^15^ The mean score across the 28 items can range from 0 to 5. The Minimal Clinically Important Difference (MCID) is 1.^16^

*Motor function (Primary outcome):* The Wolf Motor Function Test performance time (WMFT) is an in-clinic assessment of the time to complete 15 standardized tasks.^17^ It has an established reliability and validity,^17^ and has been commonly employed in previous CI therapy trials.^2^,^4^,^8^ The MCID for the WMFT is a 16% decrease in performance time.^18^ WMFT performance time scores were natural log transformed prior to analysis, following precedent,^14^ to render the data normally distributed and to approximate percentage change.

*Exploratory outcomes* included the Quality of Life in Neurological Disorders (Neuro-QoL)], 9-Hole Peg Test, tactile sense measured with the Semmes-Weinstein Monofilament test, and accelerometry.^3^ Baseline cognition (Montreal Cognitive Assessment) and adherence (e.g., minutes of active game play) were also assessed as covariates (Supplement-4.5).

### Statistical analysis

A target of 224 participants was set to obtain > 80% power to detect a 16% MCID between the groups on the WMFT.^18^ Power was estimated using a Monte Carlo approach^19^ in MATLAB with the following parameters: α = 0·05, 10% attrition, a comparative treatment effect equal to a MCID on the WMFT (estimated here using the baseline score from a previous study^2^), and within-group variability from the same study.^2^ Parallel estimation parameters projected more than adequate power (> 99%) to detect an MCID on the MAL with 224 participants.

Logistic regression examined factors related to attrition. MAL outliers (> 3 *SD* from mean) were replaced using Random Forest Multiple Imputation.^20^ Random Forest Multiple Imputation was similarly used to estimate missing data (e.g., secondary to attrition).

Both primary outcomes (WMFT and MAL) were analyzed separately via mixed effects linear models using Matlab 2020b. All participants who began treatment were included in modified intent-to-treat analyses. Treatment, time, and their interaction (the effect of interest) were entered as fixed effects, and study site and participant as random effects. *A priori* two-tailed pairwise contrasts^13^ between the experimental treatments (Self-Gaming and Tele-Gaming) and the two active comparators (CI therapy and Traditional therapist-guided self-management) were examined; Holm–Bonferroni method controlled for multiple pairwise comparisons. Several participant factors were examined as covariates to determine whether they influenced the treatment response (factor x time): baseline motor ability (WMFT), baseline arm use (MAL), adherence, tactile sense, cognition, age, gender, chronicity, and whether or not the dominant hand was more affected. See Supplement-4.8 for more detailed description of analytic procedures.

The trial was registered at ClinicalTrials.gov, NCT02631850. Data monitoring occurred during monthly research meetings by individuals who had no intellectual or financial stake in the research.

### Role of the funding source

Research reported in this article was contracted through the Patient-Centered Outcomes Research Institute (PCORI) AD-1409–20,772. PCORI did not influence the collection, analysis, or interpretation of data; writing of the report; or the decision to submit the paper for publication. The statements in this publication are solely the responsibility of the authors and do not necessarily represent the views of PCORI, its Board of Governors or Methodology Committee. All authors had access to study data, reviewed the manuscript, and consented to its publication.

## Results

### Participants and data fidelity

One-hundred ninety-three participants were recruited between Feb 9, 2016 and April 23, 2019 across the five study sites. Higher-than-expected attrition of 13% occurred prior to treatment and disproportionally at one site (OR = 3·3, *p* = 0·01, Fig. 1, eTable 7). During-treatment attrition was typical at 10%^21^ (Fig. 1). Attrition in follow-up was 25%; it disproportionately occurred at the same study site (OR = 6·0, *p* < 0·01) and for the Self-Gaming and Traditional groups that had received less frequent therapist contact (OR = 2·6, *p* = 0·01, eTables 6–7). Only one participant who remained in the study had any missing primary outcomes data (Supplement-5.5.1). Assessors remained naïve to treatment group. One follow-up and three pre-treatment MAL scores were outliers (Supplement-5.7). Post-hoc power was 78% and > 99% to detect a MCID on the WMFT and MAL, respectively, assuming between-group treatment effects equal to the MCID, 167 participants, and the variability observed in the study sample (Supplement-5.6).

### Baseline characteristics

The sample was racially and geographically diverse and generally representative of the chronic stroke population. The duration of time since stroke (chronicity) was about five years on average (Table 2, eTable 8).

### Statistical results for the primary outcomes

A modified intent-to-treat analysis assessed the effectiveness of the interventions amongst participants who began treatment, but whose adherence to the prescribed treatment was often incomplete (Table 3). Two alternate analyses using either last-observation-carried-forward imputation or no imputation reported similar results (Supplement-6, eTables 14–16). Secondary outcomes are presented in Supplement-4.5.2.

### Quality of arm use for daily activities (MAL)

**Fig. 2:**
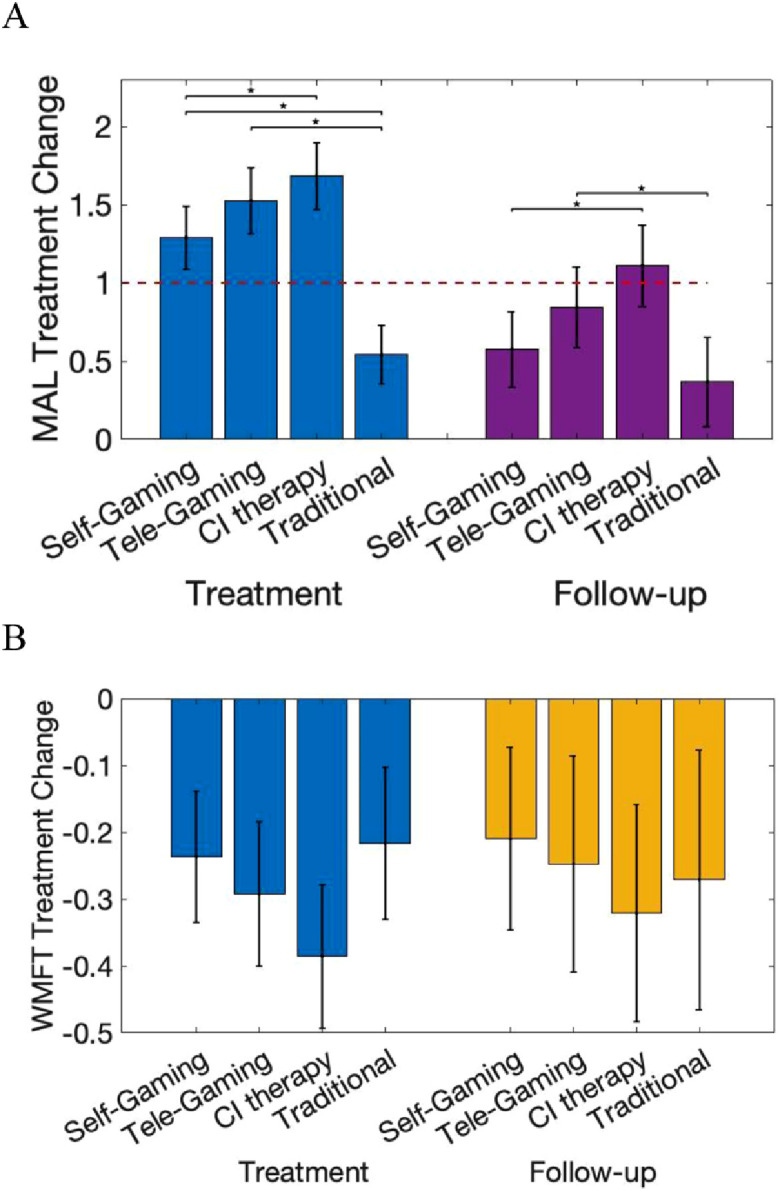
**A.** Treatment change on the MAL (change in MAL mean from baseline) by group during the intervention period (light gray, left) and 6-month follow-up (dark gray, right). MAL mean can range from 0–5 with an MCID of 1 (red dashed line). Error-bars reflect the 95% confidence interval. Statistically significant between-group comparisons are shown with an *. **B.** Treatment change on the WMFT (natural log transformed) by group during the intervention period (light gray, left) and 6-month follow-up (dark gray, right). The possible range of the natural log transformed WMFT treatment change is −4·78 to 4·78. Log differences in WMFT of −0·2, −0·3, and −0·4 log units reflect 18%, 26%, and 33% improvement, respectively. MAL = Motor Activity Log, WMFT = Wolf Motor Function Test, MCID = Minimally clinically important difference.

The gaming self-management groups that received behavioral techniques reported much greater increases in quality of everyday arm use compared to the Traditional group at post-treatment; between-group differences relative to the Traditional group were 0·8 (95% CI 0·5 to 1·0) and 1·0 (95% CI 0·8 to 1·3) for the Self-Gaming and Tele-Gaming groups, respectively (Table 3, Fig. 2A). Self-Gaming had a statistically worse outcome than CI therapy (−0·4, 95% CI −0·6 to −0·2, −24%, *p* = 0.001), but Tele-Gaming did not (*p* = 0.22). The portion of participants who achieved a clinically meaningful treatment response on the MAL was 70%, 80%, 92%, and 24% for the Self-Gaming, Tele-Gaming, CI therapy, and Traditional groups, respectively. Across all groups, participants exhibited an average 57% retention of MAL gains at 6-month follow-up; the pattern of inter-group differences at post-treatment highlighted above was largely preserved (Fig. 2A). The portion of participants who maintained a clinically meaningful treatment response was 32%, 38%, 56%, and 21% for the Self-Gaming, Tele-Gaming, CI therapy, and Traditional groups, respectively. Better motor function at baseline (WMFT) was associated with greater MAL improvement and retention, whereas higher MAL scores at baseline were associated with less improvement (eFigures 2–3, eTables 10–11).

### Standardized motor function assessment (WMFT)

All groups attained statistically significant and clinically meaningful improvements in the time to complete standardized motor tasks; median post-treatment improvements ranged from 17% to 26% of the maximum possible improvement (eFigure 4). Self-Gaming was marginally less effective than CI therapy immediately post-treatment (between-group difference in log performance time was 0·18, 95% CI 0·00 to 0·37, −46%, *p* = 0.05, Fig. 2B). The proportions of individuals who attained clinically meaningful improvements were 47%, 53%, 70%, and 45% for the Self-Gaming, Tele-Gaming, CI therapy, and Traditional groups, respectively. Reliable between-group differences were absent at follow-up. Motor gains were well maintained (Fig. 2B), with 54% of participants achieving clinically meaningful improvement through follow-up. Participants with poorer initial motor ability attained more robust improvements in motor function (eTables 12–13, eFigure 5).

### Adverse events

There were no serious study-related adverse events. Two minor study-related adverse events resolved quickly and did not affect study participation (Supplement-5.4).

### Exploratory analyses

#### Traditional group crossed over to in-home gaming

Nineteen participants completed both the cross-over intervention and post-cross-over motor testing. Mixed-effect general linear models applied to these 19 complete cases examined the main effect of time on the WMFT and MAL. Interaction terms were not included given the small sample size. Motor function improved through the post-cross-over period (pre-treatment to post-cross-over WMFT difference = −0·34, 95% CI −0·61 to −0·07, eFigure 8 left), with 68% ultimately achieving a clinically meaningful change in motor function. MAL improvements were more modest (pre-treatment to post-cross-over gains =0·84, 95% CI 0·51 to 1·17), with only 37% having achieved clinically meaningful improvement post-crossover (Supplement 5.12).

#### Adherence

Most participants completely adhered to the therapist-led portion of the treatments, whereas adherence to self-managed treatment was variable (Supplement 5.11). The Tele-Gaming group, which experienced more frequent therapist contact, had significantly better adherence to in-home gaming than the Self-Gaming group (one-tailed Wilcoxin rank sum test, *z* = 1·74, *p* = 0·04). The Self-Gaming group completed a median of 7 h of the prescribed 15 h (46%) of gaming practice, while the Tele-Gaming group completed a median of 12 h (81%, eFigure 6). Twenty-four percent of Self-Gaming participants and 37% of Tele-Gaming participants were fully adherent (≥ 15 h), but an approximately equal portion adhered very poorly (36% and 22%, respectively), ultimately completing less motor practice than the Traditional group. Median adherence to the CI therapy restraint mitt was 62%. Neither duration of motor practice nor mitt use significantly influenced extent of improvement on either primary outcome (Supplement 5.11, eFigure 7).

## Discussion

The VIGoROUS trial is the largest trial of self-management for neurological recovery completed to date. It is also the first randomized controlled trial to evaluate the effectiveness of a flipped model of care in which therapist time is allocated towards behavioral intervention and motor practice is entirely self-managed through video games. Given the pragmatic nature of the trial, the sample is racially/geographically diverse, had a mean chronicity at enrollment representative of those living with long-term effects of stroke,^22^ and includes participants with comorbidities and various stroke etiologies. In accordance with the hypothesis, intent-to-treat analyses showed that the flipped model of care produced greater improvements in quality of everyday arm use (MAL) than Traditional therapist-led motor rehabilitation. Of the two gaming interventions, the Tele-Gaming intervention that provided more extensive behavioral support yielded improvements on both primary outcomes that were comparable to CI therapy, while using just a fifth as much therapist time.

Contracting, self-assessment, and problem-solving are behavioral techniques that help clients reflect on how they use their paretic arm for daily activities and reduce over-reliance on the stronger arm. A small prior RCT showed that behavioral techniques greatly enhance the extent to which treatment-induced motor gains translate into improved arm use during daily activities, irrespective of motor training modality.^2^ This trial additionally showed that behavioral techniques can improve use even when motor training is entirely self-managed. Absent these behavioral techniques (e.g., Traditional group in this trial), quality of daily arm use (MAL) does not improve meaningfully despite motor gains (i.e., faster WMFT performance), consistent with prior reports.^2^,^3^ As the ultimate goal of rehabilitation is to increase functional use of the paretic arm and hand, there appears to be a marked disconnect between the techniques that can best accomplish this goal and current rehabilitation practice. The model of game-based self-management tested here can effectively expand use of behavioral techniques by freeing up therapist time that is normally dedicated to supervised motor practice.

Of the two gaming protocols examined here, the one that added brief twice-weekly telerehabilitation behavioral consults was more successful at promoting and maintaining improved quality of paretic arm use during daily activities (Fig. 2). Moreover, quality of daily arm use did not improve meaningfully for most participants crossed-over to a gaming protocol that required them to self-administer the behavioral techniques (Supplement-5.12), consistent with prior work on self-managed behavioral intervention.^10^ Taken together, the results suggest that therapist dialog and/or accountability plays a critical role in driving behavior change. Additionally, therapist contact was critical for promoting adherence to self-managed motor practice, as reflected in a median increase of 52 additional minutes of game play from each brief teleconsultation. This suggests that brief, but frequent, therapist feedback/problem-solving (uniquely feasible through telehealth), may be the most efficient and cost-effective way to support self-management, improve adherence, and enhance behavior change.

Consistent with prior work,^8^,^10^,^12^,^14^,^23^ all four models of care produced similar improvements in time to complete standardized motor tasks (WMFT) even though they involved markedly different doses and modalities of motor practice. The robust and sustained improvements (∼20% of maximum possible improvement, Fig. 2B, eFigure 4) demonstrate that the majority of individuals who are nearly 5 years post-stroke on average can achieve clinically-meaningful improvements in motor capacity,^24^ even with as little as 5 h of therapist intervention. Results are consistent with prior evidence that gaming can be as effective as traditional modalities, ^12^,^23^,^25^,^26^ and additionally demonstrate its efficacy as a self-management approach. Motor practice through gaming may be more efficient than traditional self-management programs (e.g., booklet exercises and task practice), which have either shown modest unsustained improvements^27^ or have employed much more intensive protocols to achieve similar improvements.^10^,^28^ As self-managed game-based rehabilitation is effective and uses the same principles of therapeutic progression employed by therapists, gaming should ideally be utilized outside of therapist consultations to increase the overall intensity of motor practice, rather than being directly supervised by therapists. Without such a strategy to reduce therapist effort, the cost advantages of tele-health delivery are modest.^8^

The gaming modality offers several additional practical advantages to therapist-led motor practice. It allows automatic progression of difficulty (eTables 4–5) and can be done largely independently in the home, making it more accessible and well suited to telerehabilitation. Additionally, rehabilitation gaming systems continuously and remotely track motor improvement,^11^ effort (e.g., range of motion, reps per time), and adherence. Gaming can also be an engaging way of delivering unlimited motor practice at a low one-time cost. This can enable individuals with more severe motor impairments to receive the >90 h of intervention required to achieve clinically meaningful improvement,^29^,^30^ and for all stroke survivors to reduce the dire adverse health impacts of post-stroke sedentary behavior.^31^ Finally, self-management through gaming enables therapists to devote scarce treatment time to behavioral interventions that cannot be effectively self-administered.

As was expected for a pragmatic trial in self-management, adherence to in-home gaming rehabilitation was imperfect, particularly for the Self-Gaming group that received fewer therapist consultations. Thus, this trial does not provide a dose-matched comparison of the efficacy of game-based versus in-clinic motor practice (such is published elsewhere^12^). Given that 36% of Self-Gaming and 22% of Tele-Gaming participants completed less than one third of the prescribed self-directed motor practice, adherence-enhancing interventions targeting this vulnerable subset of the stroke population should remain a priority for future research.

Limitations to internal validity include reduced power (78%) to detect between-group differences on the WMFT due to not meeting the recruitment target, not assessing how baseline motivation may factor into response to treatment, and inability to reliably measure adherence to self-managed portions of the interventions that did not involve technology (e.g., home exercises and task practice). Follow-up data should also be cautiously interpreted given substantial and unequal attrition. The extant data suggest that “tune-up” sessions may be required to sustain MAL improvements long-term, given 57% retention of gains 6 months later. Follow-up data may additionally underestimate the long-term effects that can be achieved through gaming self-management, as participants did not retain access to the gaming systems during the follow-up period.

The comparative treatment effects observed here may only generalize to individuals treated in an outpatient setting who have some movement in both the proximal and distal upper extremity; this reflects nearly half of all individuals living with upper extremity paresis (Fig. 1). Supplement-7 describes additional Limitations. Nonetheless, poor generalization of rehabilitation gains to daily life remains a universal challenge in neurologic rehabilitation; thus, the self-management behaviorally-focused model of care proposed here could be broadly applied across multiple interventions and populations.

In conclusion, self-managed motor practice through gaming with therapist-guided behavioral intervention is as effective at improving motor capacity and more effective at improving quality of everyday arm use than traditional therapist-supervised motor rehabilitation. Moreover, a self-management approach that employs brief telehealth behavioral consultation produces outcomes similar to in-clinic CI therapy, but requires just one fifth as much therapist time. Results of this pragmatic trial support the need for a marked shift towards prioritizing behavioral intervention during neurologic motor rehabilitation, as upper limb motor practice can be effectively self-managed at home.

## Contributors

LVG directed the study, served as site PI for OSU until Aug 2018, completed the analyses with support from statistical consultants, and drafted the manuscript. DSNL served as site PI for OSU and administrative PI for the study after LVG transferred to a different institution; she managed the study data, provided edits to the manuscript, and reported study results to clinicaltrials.gov. GU served as site PI for UAB, verified the statistical analysis and results reporting, and provided edits to the manuscript. NS, a stroke survivor, dialogued with the stroke community and scientist-investigators to select comparators and outcome measures that felt most relevant to stroke survivors, drafted some of the treatment forms, and served as site PI for PMMC. MS served as site PI for OhioHealth. ET informed the treatment protocol for the CI therapy arm and provided edits to an earlier draft of the manuscript. RP served as site PI for Missouri U and provided edits to the manuscript. KK supervised the majority of study treatments and provided edits to the manuscript. DM and AB drafted the Traditional protocol based on input from several practicing occupation and physical therapists; AB provided minor edits to the manuscript. RC directed technical support and in-game data collection. LPL provided oversight of treatment fidelity. VWM established the medical inclusion/exclusion criteria, provided medical oversight for the trial, and provided minor edits to the manuscript. All authors had full access to all study data and had final responsibility for the decision to submit for publication.

## Declaration of interests

Drs. Gauthier, Borstad, Lowes, and Crawfis co-founded Games That Move You, PBC to commercialize the gaming technology utilized in this research; conflict management plans were put in place through The Ohio State University prior to conducting this trial to ensure research integrity.

## Appendix Supplementary materials

Image, application 1
