# Supplementary material for: Video game rehabilitation for outpatient stroke (VIGoROUS): A multi-site randomized controlled trial of in-home, self-managed, upper-extremity therapy
Source: eClinicalMedicine. 2021 Dec 17;43:101239. doi: 10.1016/j.eclinm.2021.101239 (PMC8688168; doi:10.1016/j.eclinm.2021.101239)
Supplement: Supplementary file 1 [file mmc1.docx]

**Supplement**

1. Protocol 2

2. Amendments to the Analytic Plan 2

3. e-Introduction 3

**eTable 1: Behavioral techniques to promote increased arm use during daily activities.** 3

4. e-Methods 4

4.1 Recruitment 4

**eTable 2: Participant recruitment** 4

4.2 Randomization Procedure 4

4.3 Interventions 5

**eTable 3. Treatment elements of the four interventions.** 5

**eFigure 1: Screenshots of Recovery Rapids.** 6

**eTable 4: Movements trained with the gaming system.** 7

**eTable 5: Principles of motor learning underlying the development of Recovery Rapids** 7

4.4 Treatment fidelity 9

4.5 Assessment 9

**4.5.1** **Primary Outcomes** 9

**4.5.2** **Secondary/exploratory outcomes:** 10

4.6 Time frame for the study 11

4.7 Procedures to maximize adherence and retention 11

4.8 Analytical and statistical approaches: 11

4.9 Regulatory 13

5. e-Results 13

5.1 Recruitment 13

**eTable 6: Number screened and enrolled by site and reasons for exclusion.** 13

5.2 Participants 13

5.3 Attrition 14

**eTable 7. Characteristics of participants by attrition status** 14

**eTable 8: Enrollment and attrition by site.** 14

5.4 Adverse Events 15

5.5 Missing data 15

**5.5.1** **Primary Outcomes** 15

**5.5.2** **Exploratory Outcomes** 15

5.6 Post-hoc power calculation 16

5.7 Outlier adjustment 16

5.8 Tests of Normality 16

5.9 Supplemental MAL results 17

**eTable 10: General linear mixed effects model for the MAL Model ^a^** 17

**eTable 11. Effect sizes for the patient characteristics associated with MAL response** 17

**eFigure 2: Gains in arm use versus baseline motor ability.** 17

**eFigure 3: Gains in arm use versus baseline arm use.** 18

5.10 Supplemental WMFT Results 18

**eFigure 4: Boxplot of proportional improvements by group on the WMFT.** 18

**eTable 12: General linear mixed effects model for the WMFT primary outcome:** 19

**eTable 13· Effect sizes for the patient characteristics associated with WMFT response.** 19

**eFigure 5: Gains in motor ability versus baseline motor ability.** 19

5.11 Adherence 19

**eFigure 6: Boxplot of adherence to game play** 20

**eFigure 7: Per-protocol analysis of the WMFT** 21

5.12 Traditional group crossed-over to Gaming self-management. 21

**eFigure 8: Continued improvement after 6-month cross-over to gaming.** 22

5.13 Analysis of Secondary Outcomes 22

6. Supplemental Statistical Analysis 23

6.1 Intent-to-treat analysis as originally planned in the published study protocol 23

**eTable 14. Descriptive Statistics of Supplemental Intent-to-treat analysis.** 23

**eTable 15: Comparative treatment effects for Supplemental Intent-to-treat analysis.** 24

6.2 Analysis of only existing data, no imputation performed. 24

**eTable 16. Analysis without imputation.** 24

7. Limitations 24

7.1 Limitations to external validity: 24

7.2 Limitations to internal validity: 25

8. References 27

# Protocol

A descriptive protocol for this trial was peer reviewed and published in BMC Neurology in 2017.^1^ The full treatment protocol, including all study forms, can be found here: https://studentuml-my.sharepoint.com/:w:/g/personal/lynne_gauthier_uml_edu/ESXH1X7a-FJDohlbCM4R2PUBJpOTrhp9maz6Mg5I-7seWg?e=gRrXlh

# Amendments to the Analytic Plan

For the intent to treat analysis assessing comparative effectiveness, imputation via Random Forests Interpolation (RFI, see below), rather than list-wise deletion, was employed to account for missing data at post-treatment. This modification was made for 2 reasons: 1) because list-wise deletion and last-observation carried forward methods have been shown to produce biased parameters and estimates and 2) because imputation was originally proposed for the analysis of data that is missing at follow-up, employing it throughout maintains greater consistency. For comparison, the results of the analysis that was originally planned in the study protocol are reported in eTables 13 & 14 of the Supplement.

While the published protocol mentioned testing for outliers as a data quality control procedure, it did not include a detailed plan to address outliers during data analysis. Given that the Motor Activity Log (MAL) is a patient-reported measure, and that outliers may reflect poor memory or awareness in a population where cognitive impairment is prevalent, we decided to interpolate outliers for the MAL via RFI. Outliers would only be interpolated for the objective measures (e.g., Wolf Motor Function Test) in cases of assessor error (e.g., broken standardization).

The published protocol incorrectly identified the Neuro-QoL as a primary outcome measure in Table 4. It was intended as a secondary outcome measure per the text of the published protocol and the description on clinicaltrials.gov. Accordingly, it was analyzed as a secondary outcome measure.

The Brief Kinesthesia test was dropped as a potential covariate (i.e., participant factor that may influence treatment response) because it did not appear to measure proprioceptive ability as originally intended (was unreliable and confounded by motor ability).

Accelerometry data was captured via using the PebbleTime smart watch in lieu of wearable sensors that combined an accelerometer and magnetometer to quantify shoulder, elbow, and trunk angles. This change occurred after several sensors that the team tested either failed to yield reliable movement data or lacked at least 8 hours of battery life.

When examining participant characteristics that influence treatment response, baseline motor function was analyzed as a continuous variable using the baseline score on the WMFT, rather than a dichotomous variable (ability to place any pegs on the 9-hole peg test, as originally proposed). Continuous variables provide more information about the construct of interest than dichotomous variables do.

# e-Introduction

**eTable 1: Behavioral techniques to promote increased arm use during daily activities.** The treatment materials for this study (i.e., to implement these techniques) can be found at the following URL: <https://drive.google.com/open?id=0B87rU40WPJFKcHdYdkdlNTJpWWs>. For more detail on these behavioral approaches, see Morris, D., Taub, E., & Mark, V. (2006)^2^, the published study protocol,^1^ and an educational Youtube video.^3^

| **Technique** | **Description** |
| --- | --- |
| Goal setting | Participants identify 3 meaningful goals that can be accomplished within the treatment period. They rate the importance of each goal on a 1-10 scale. Therapists solicit motivational statements regarding the importance of each goal (e.g., the statement “What led you to rate that goal an 8/10 in importance and not a 3/10?” encourages the participant to explain why the goal is important to him/her). |
| Treatment Contract | Participants complete a signed agreement outlining expectations for the treatment, including agreement to use the weaker arm during all daily activities in which it is safe to do so. Each activity in a participant’s daily routine is then broken down into its detailed component parts (e.g., open drawer, transport spoon to table, open fridge, get milk, open milk, pour cereal, pour milk, eat with spoon). This results in a list that typically contains over 100 component activities, along with a mutually agreed-upon expectation of how the weaker arm could be used during each activity. Multiple copies of the activities list from treatment contract are provided so that the participant can track arm use daily (see Self-monitoring). |
| Caregiver Contract | Caregivers agree to provide encouragement and opportunities for the participant to attempt daily tasks with the weaker arm. Involved caregivers are provided with some basic training in guided problem-solving to support the participant’s behavior-change efforts at home. |
| Self-monitoring | Participants check off which activities from the treatment contract were completed at the end of each day. This log is then reviewed with the therapist at the following session. Participants also wear a smart-watch on the paretic arm during waking hours that provides vibratory feedback and a “please use me” notification when periods of inactivity are detected. |
| Guided Self-monitoring | Participants self-rate performance on a subset of these daily activities via informal therapist administration of a rotating subset of items on the Motor Activity Log (MAL). The MAL is a self-assessment of the amount/quality of arm use for 28 universal activities of daily living (the majority of which represent ADLs that would also be present on the activities list from the Treatment Contract). Informal administration of the MAL prompts the participant to think more deeply about how the weaker arm was used during some of the activities listed on the treatment contract and has a therapeutic effect.^4^ |
| Problem-solving | Therapist prompts participants to self-identify potential solutions to barriers interfering with use of the paretic arm during guided self-monitoring or while reviewing participants’ activity logs. Therapist documents an action plan describing participants’ chosen strategies. If the participant reports poor participation with the weaker arm for many different activities, therapist will prioritize problem-solving through activities that relate most strongly to client goals. |
| Home Practice | Participants agree to repetitively practice specific tasks that align with their treatment goals for a target of 30 minutes per day using the weaker arm (e.g., placing a golf ball on the ground and putting a golf ball into a can if their goal is to resume playing golf). |

# e-Methods

## **Recruitment**

### **eTable 2: Participant recruitment**

| **Recruiting sites** | - The Ohio State University - academic medical center serving ~50% rural clientele, including Apalachia - OhioHealth – outpatient neurorehabilitation clinic serving ~50% rural clientele - University of Alabama Birmingham - academic medical center serving a large African American clientele - Providence Medford Medical Center - outpatient neurorehabilitation clinic serving mainly rural clientele - Missouri University - academic medical center serving primarily rural areas in central Missouri |
| --- | --- |
| **Method of recruiting** | At the three academic medical centers, prospective participants were identified primarily through mining electronic medical records for ICD-9 and ICD-10 codes for stroke and hemiparesis or through searching opt-out medical center databases for potentially eligible participants. Potential participants then received a letter informing them of the study (OSU) or received a phone call (UAB, Missouri). Potential participants at the two outpatient clinic sites were primarily identified through referrals from therapists and physicians. To lessen the burden on potential participants, study staff offered a brief phone screening. Participants that appeared to meet eligibility criteria based on the phone screening were then invited to participate in an in-person screening session to ensure that they met the full inclusion criteria prior to being enrolled in the study. |
| **Inclusion criteria** | - At least 6 months post-stroke - Experienced at least 1 stroke of any etiology - Mild to moderate upper extremity hemiparesis in which the following motor criteria below are met: > 10° active range of motion in at least 2 fingers, thumb, and wrist; > 45° active range of motion for shoulder abduction and flexion; > 20° elbow extension from a 90° flexed starting position - Ability to provide informed consent - Expressed willingness to comply with all study procedures and attend all study-related visits. - Age ≥ 18. - Ability to follow one-step commands. - Community-dwelling with transportation to therapy sessions. - Ability to operate the gaming system with minimal assistance, including sufficient corrected vision to perceive game objects from a distance of 5 feet. |
| **Exclusion criteria** | - Concurrent participation in other experimental upper extremity rehabilitation trials. - Concurrent participation in other outpatient rehabilitation for the upper extremity during the treatment phase(s) of the study. - Upper extremity Botox within 3 months prior to beginning study-related treatments - Already exhibiting substantial use of the more-affected arm in daily life (Motor Activity Log [MAL] mean score at screening > 2.5). - Major medical conditions that would render intensive rehabilitation infeasible or unsafe. - Had received CI therapy previously. |

## **Randomization Procedure**

Participants were initially stratified by motor ability prior to randomization by a research assistant or study coordinator. Stratification was based on the participants’ ability to place any number of pegs on the 9-Hole Peg Test within 120 seconds. The 9-hole Peg Test was chosen as the instrument to determine stratification because it could be rapidly administered by a research assistant during the in-person screening visit. This procedure increased the likelihood of achieving balanced initial motor ability across study groups. Participants stratified as having greater motor ability (able to place at least 1 peg) randomly drew a folded sticky note or small opaque envelope containing their group assignment from one large opaque envelope, while those with greater disability drew from a separate large opaque envelope. This randomization procedure was selected because “drawing from a hat” was more trusted by the stroke community than computer-generated randomization and provided participants with a sense of greater autonomy during the randomization process (i.e., they “picked” their own group at random rather than being assigned a group by study personnel). Both the experimenters and participant were naïve to treatment condition prior to randomization (concealed allocation).

## **Interventions**

*Timeline*: Treatments are outlined in eTable 3 and described in more detail below. All treatments were scheduled over a 3-week period, which could be extended to 4 weeks in the case of illness or other unanticipated conflicts/events.

*Co-occurring interventions*: None of the enrolled participants participated in other upper extremity treatments during the active treatment phase of the study. Being several years post-stroke on average, most had not received any outpatient therapies recently. Participants were free to engage in other therapies during the follow-up period, but most did not. Gaming participants did not retain access to the game during the follow-up period.

**eTable 3. Treatment elements of the four interventions.** M = Hours of prescribed active motor practice with feedback and real-time progression (therapist-led or gaming). H = Hours of prescribed home exercises (no feedback or real-time progression). B = Number of therapist consultations addressing behavioral techniques to promote adherence and use of the weaker arm during daily activities. T = Number of therapist contacts.

| **Treatment** | **Therapist supervised motor practice** | **Behavioral intervention**  **to promote increased daily use of the weaker arm** | **Home practice prescription** | **M** | **H** | **B** | **T** |
| --- | --- | --- | --- | --- | --- | --- | --- |
| **Self-Gaming**   - motor and behavioral intervention - # therapist contacts = traditional group | Therapist teaches game play in-clinic for <30 minutes on day 1. | - 4·5 h in-clinic over 4 d: Contracting, Problem-solving, Goal setting. - Self-assessments of arm use at home through computer program (3 min x 10 d), reviewed during treatment sessions | - 15 h of motor practice via Recovery Rapids game (self-paced over 3 w) - 30 min targeted practice on ADLs related to participant goals x 10 d - Encouraged to use weaker arm for specific daily activities. | 15 | 5 | 4 | 4 |
| **Tele-Gaming**   - motor and behavioral intervention - # sessions behavior intervention = CI therapy - time in-clinic = traditional group | Therapist teaches game play in-clinic for <30 minutes on day 1. | - 4·5 h in-clinic over 4 d: Contracting, Problem-solving, Goal setting. - Self-assessments of arm use at home through computer program (3 min x 10 d), reviewed during treatment sessions - 6 video consultations focused on problem-solving, totaling 2·6 h | - 15 h of total motor practice via Recovery Rapids game (self-paced over 3 w) - 30 min targeted practice on ADLs related to participant goals x 10 d - Encouraged to use weaker arm for specific daily activities. | 15 | 5 | 10 | 10 |
| **In-clinic CI therapy**   - motor and behavioral intervention | 15 hours active motor practice over 10 d | - In-clinic ~5 hours over 10 d: Contracting, Self-assessment, Problem-solving, Goal setting. | - 30 min targeted practice on ADLs related to participant goals x 10 days. - Encouraged to use weaker arm for specific daily activities. | 15 | 5 | 10 | 10 |
| **Traditional**   - motor intervention | 5 hours active motor practice over 4 d | - General encouragement to use the weaker arm more at home. | - 30 min home strengthening program based on GRASP^5^ x 10 d | 5 | 5 | 0 | 4 |

*Gaming CI therapy with self-management (Self-Gaming):*

This treatment followed a brief time-limited treatment schedule in accordance with it being a therapist-guided self-management program. The intensity of in-clinic treatment was typical of most standard clinic settings (e.g., one to two hours per week), but the duration was shorter, reflecting patient preference for a compressed therapy schedule (just 3 weeks). Therapist consultations emphasized the behavioral techniques of CI therapy instead of motor practice. Per the principles of CI therapy, participants were tasked with completing 15 hours of active motor practice with just the paretic arm through gamified self-management.

*About the game:* Given that many stroke survivors have insufficient access to rehabilitation, the intervention for this group was designed to empower stroke survivors to manage their own care. A common challenge reported by stroke survivors and therapists alike is a difficulty adhering to self-managed home practice programs. Computerized gaming interventions are preferred by stakeholders and have established efficacy,^11^ therefore a custom kayak-adventure-themed rehabilitation game, Recovery Rapids,^12-14^ provided a more palatable vehicle for intensive practice at home.

Recovery Rapids uses the Microsoft Kinect v2 sensor to capture 9 proximal and distal movements made by participants that drive the navigation of an avatar through various obstacles. The Microsoft Kinect v2 sensor identifies a “skeleton” consisting of X, Y, Z coordinates of 17 upper body joints at 15-30 Hz, from which joint angles are calculated. The skeletal reconstruction is shown in the bottom two images of eFigure 1. “Joints” include the thumb tip, tip of the most extended finger, hand, wrist, elbow, and shoulder of each arm; upper, middle, and base of the spine; head; and neck. A movement is detected when the player progresses through a series of body positions in sequence and while staying within certain constraints. For example, shoulder abduction is registered when the more impaired arm is first at the player’s side (shoulder angle in the coronal plane is less than 20 degrees from vertical), then lifted to the side such that the shoulder angle in the coronal plane exceeds a certain threshold, all while the elbow angle remains above a certain threshold. Consistent with in-clinic treatments, the game automatically progresses the required difficulty of movements as a person improves using a performance-based algorithm (eTable 4). For example, the difficulty of shoulder abduction increases (greater shoulder range of motion and elbow extension is required to trigger a movement) until the player achieves about 80% success. Each movement is mapped to an intuitive game action (eTable 4). Recovery Rapids was designed around several principles of motor learning (eTable 5). In keeping with CI therapy’s constraint of the stronger arm, only movements made with the more affected upper extremity can trigger game actions. eFigure 1 shows screenshots of the game.

To keep game play novel, Recovery Rapids was designed using procedural content generation, which means that the gaming environment is generated “on the fly” as a person plays so that obstacles change their type and position. This allows the game to generate the desired balance of hand, wrist, elbow, and shoulder activities specified by the therapist. The game also has minigames embedded within for greater variety. Each successfully executed movement and avoidance of an obstacle scores points, while collisions with obstacles cost the player points. Additional detail on game design and screenshots of the game can be found in prior publications.^12-14^

**eFigure 1: Screenshots of Recovery Rapids.** The user pilots a kayak downstream while avoiding obstacles and collecting treasure. Game actions are triggered by movements of the paretic arm.


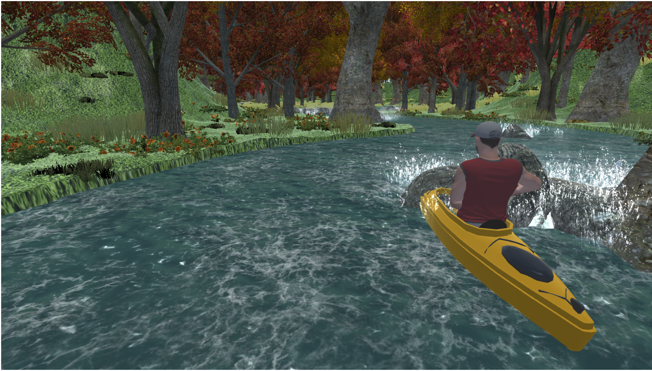

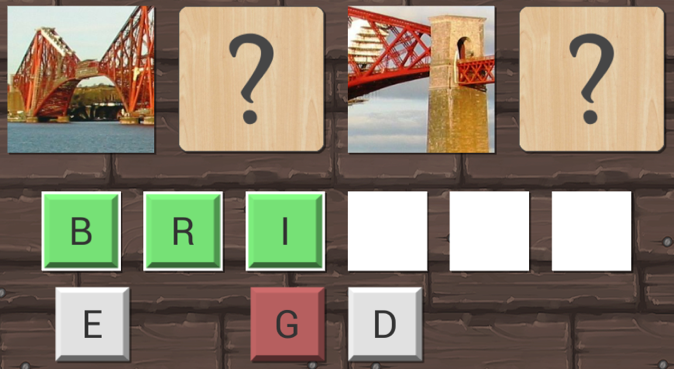

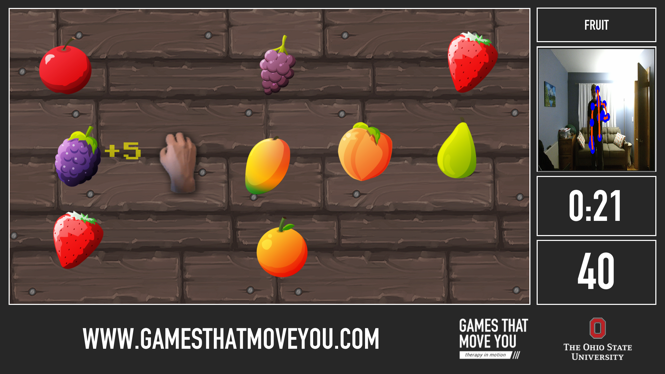

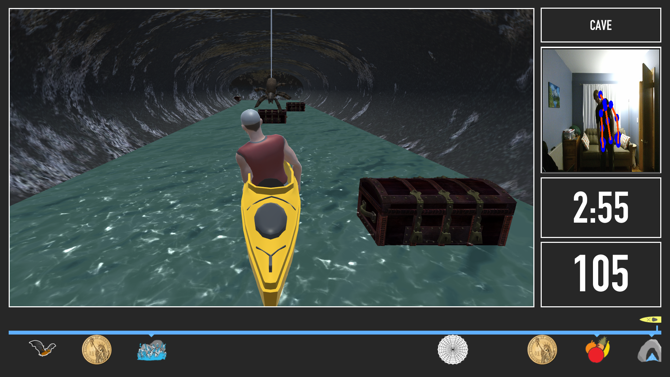


*Motor practice:* Participants agreed to play Recovery Rapids for a total of 15 hours (1·5 hours per day) on 10 treatment days over three weeks. This treatment schedule was designed to dose-match the duration of *active* motor practice provided in CI therapy (the same duration of active motor practice could be accomplished in half of the time because gaming treatment involves continuous practice without breaks for task set-up; automated feedback also occurs concurrently with game play). Participants could self-pace their game play (i.e., pause for short rests or begin another session later). Participants were encouraged to break play into 3 separate sessions per day to avoid fatigue, but ultimately had the freedom to set the frequency and duration of their sessions. Participants self-scheduled their gameplay. They were asked to make up any missed play time on a non-treatment day to encourage adherence to the prescribed motor practice. Game play was driven solely by movements made with the more affected upper extremity. The gaming system logged compliance with in-home game play (i.e., *active* play time).

*Behavioral techniques:* A total of 5 hours of in-clinic therapist consultation was delivered over 4 treatment sessions (initial session = 2 hours). This treatment schedule was chosen because the frequency of in-clinic visits (1-2 times per week) reflects that of routine clinical practice. Therapist sessions focused on teaching game play (about 30 minutes in session 1) and on delivering the same CI therapy behavioral techniques described in eTable 1 and elsewhere.^2,3^ Behavioral techniques consisted of goal setting, a treatment contract, caregiver contract (when a caregiver was present and available), daily self-monitoring of use of the weaker arm during activities of daily living (ADLs), guided problem-solving to overcome barriers interfering with use of the weaker arm, and 30 minutes/day x 10 days of prescribed home practice on activities of personal importance (e.g., those that are relevant to the participant’s goals). Due to the shortened therapist contact time, a brief computerized self-assessment of arm use was self-administered at home on the gaming system and responses were reviewed at the next therapist meeting.

*Reminders to use the paretic arm:* In lieu of the CI therapy restraint mitt (a component of CI therapy that is not well liked by participants), participants were provided with a SmartWatch application that tracked use of the weaker arm and notified the participant when prolonged inactivity was detected (vibration with message to “Please use me”).

*Follow-up check-ins:* In the month following treatment, continued use of the weaker arm during daily activities was encouraged by prompting (e-mailing) the participant weekly to self-assess daily arm use via a REDCap survey.

**eTable 4: Movements trained with the gaming system.** The game automatically adjusts the active range of motion required to trigger the relevant game action to permit the participant to maintain an 80% success rate. The table below (from left to right) outlines the therapeutic movements that are prompted by the game, the parameters used by the gaming system to adjust difficulty, and the resulting game action from successfully executing a movement.

| **Task** | **Game automatically progresses** | **Game action** |
| --- | --- | --- |
| Shoulder flexion/extension with elbow extension | Shoulder flexion angle, elbow extension angle | Propel kayak forward |
| Shoulder abduction with elbow extension | Shoulder abduction angle, elbow extension angle | Steer kayak toward hemiparetic side |
| Horizontal shoulder adduction across midline | Lateral distance across midline | Steer kayak away from hemiparetic side |
| Elbow flexion/extension | Angle between hand shoulder vector and the vertical axis. | Catch fish with a net |
| Elbow flexion/extension and grasp/release | Elbow extension angle, finger extension (distance between finger tip and palm) | Collect bottles from a river |
| Forearm supination with shoulder flexion and elbow extension | Shoulder flexion angle, elbow extension angle, forearm supination angle | Catch parachute to receive supplies |
| Finger flexion/extension and thumb abduction/adduction with targeted reaching | Finger extension | Grasp and release virtual objects |
| Forearm supination with targeted reaching | Forearm supination angle | Flip over card |
| Wrist extension with targeted reaching | Fingertip to palm vertical vector. | Select letters in word puzzle |

### **eTable 5: Principles of motor learning underlying the development of Recovery Rapids**

| **Motor Learning Principle** | **How Implemented** |
| --- | --- |
| Functionally relevant task practice^15,15-19^ | A 3-dimensional virtual environment prompts complex motor patterns characteristic of real-world motor demands (eTable 4) |
| Variable practice: random, rapid alternation in task demands and complexity^15,20-22^ | Participants must switch rapidly between movements to successfully navigate the virtual world. |
| High repetition practice^23^ | Game prompts > 400 repetitions per hour. |
| Difficulty progresses according to improvement ^2,22^ | Difficulty adjusts automatically to maintain 80% success rate. |
| Implicit feedback^24,25^ | Game events only trigger when movement mechanics are of reasonable quality (relative to the player’s ability). |
| Action observation/motor imagery^26-28^ | An avatar (person in a kayak) mimics players’ movements. |
| Constraint/ forced use^29^ | Only movements of the weaker arm/hand trigger game actions. |
| Multi-modal feedback provided concurrently to more than one sensory pathway^30^ | Game actions and a sound effects co-occur with successful movement attempts. |

*Gaming CI therapy with additional teleconsultation (Tele-Gaming):*

####

The treatment administered to this group was identical to the Self-Gaming CI therapy group, but with 6 additional video conference visits to match the number of sessions of behavioral intervention that occur during in-clinic CI therapy. An initial 1-hour video consultation occurred between the first two in-person sessions, with subsequent 20-minute check-ins occurring throughout treatment (2·6 hours total video consultation). In the month following treatment, continued use of the weaker arm during daily activities was encouraged through phone administration of the Motor Activity Log by a therapist, consistent with the in-clinic CI therapy group.

*In-clinic CI therapy (high-intensity comparator):*

The in-clinic CI therapy group received all treatment elements from an established CI therapy protocol.^2^ Participants randomized to this group received ten 3.5-hour sessions of in-clinic traditional CI therapy with a therapist (35 hours total). A target 15 hours was devoted to active motor practice. Behavioral intervention was conducted every session to help translate this motor practice into everyday activity (eTable 1), encompassing about 5-7 hours of the treatment time. Breaks, feedback, and task set-up comprised the rest (~40%) of session time.

*Motor practice* consisted of five to nine tasks per day involving repetitive manipulation of real objects. Most tasks involved both proximal and distal movements. When possible, tasks represented portions of a functional task that the participant desired to improve upon. For example, if a participant wanted to drive; the participant might repetitively practice turning a wheel. Tasks were selected to be moderately challenging, but always feasible, for the participant to complete. When a participant demonstrated improved performance on a task, its difficulty was increased (e.g., a target was moved farther away to require greater range of motion to complete the task). Each task involved up to ten 30–120 second trials. Trial-level performance feedback (how quickly a task was accomplished or how many repetitions of a task were accomplished within a 30 second trial) was provided following every trial. Verbal encouragement (e.g., “Great effort!”) or coaching (e.g., “Now try a pincer grasp”) was provided for at least 80% of trials.

*Behavioral techniques:* CI therapy participants received behavioral intervention on every treatment day that was designed to help them apply motor gains made in the clinic to everyday activities at home (eTable 1 details the specific techniques). Behavioral techniques consisted of goal setting, a treatment contract, caregiver contract (when a caregiver was present and available), daily self-monitoring of use of the weaker arm during activities of daily living (ADLs), guided problem-solving to overcome barriers interfering with use of the weaker arm, and 30 minutes/day x 10 days of prescribed home practice on activities of personal importance (e.g., those that are relevant to the participant’s goals). Behavioral intervention was the same as that provided to the gaming groups.

*Restraint mitt:* CI therapy participants were also prescribed a restraint mitt to be worn on the stronger arm for a target of 10 hours daily. The purpose of the restraint mitt was to discourage use of the stronger arm during daily activities.

*Follow-up check-ins:* In the month following treatment, continued use of the weaker arm during daily activities was encouraged through phone administration of the Motor Activity Log.

*Traditional therapist-supervised rehabilitation (matched for in-clinic therapist contact):*

The treatment protocol was established by community therapists to incorporate an array of activities typically provided during outpatient upper extremity therapy between self-managed home exercises carried out by the participant. It followed a treatment schedule that is typical of standard clinic settings (e.g., once to twice weekly therapy sessions). Following a 25 min evaluation on Day 1, the following components of the intervention were delivered: 1) neuromuscular reeducation (20 min on Day 1, 10 min daily thereafter), 2) functional training (25 min all treatment days), 3) progressive strengthening (25 min on Day 1, 15 min thereafter), and 4) review/adjustment/teaching of home program (25 min on Day 1, 10 min thereafter). For the active procedures of neuromuscular reeducation, functional training, and progressive strengthening, the target for exercise intensity was 4 (somewhat hard) on the Borg CR10 Rating of Perceived Exertion Scale.^6^ A self-management home program was designed for all participants on the first visit, modeled on the strengthening exercises from the GRASP self-management program^5,7^ and the LEAPS trial.^8^ This home program consisted of strengthening exercises (including use of Thera-Band when appropriate) to be done for 15 min twice daily (total of 30 min daily) on the first 10 non-therapy days. Strengthening home exercises are commonly employed in traditional care because they are inexpensive, accessible, easy to teach, and their use is generally supported in the literature.^7,9,10^ The intensity of self-managed motor exercises (15 minutes twice daily) was chosen because this is the duration of practice that therapists working in outpatient neurologic rehabilitation settings indicated that they would prescribe to their clients and it is consistent with the duration of behavioral “homework” that was assigned to the other treatment groups.

After the 6-month follow-up, the Traditional group received the opportunity to cross over to a completely self-managed gaming intervention. This self-managed intervention involved a single two-hour therapist consultation to teach game play and orient the participants to the behavioral approaches. Participants were then tasked with completing 15 hours of game-based motor practice at home and periodically completing behavioral change treatment forms; they self-managed both the gaming practice and behavioral interventions without additional therapist support. This cross-over was mainly included for ethical reasons (i.e., so that all participants could have the opportunity to receive a different and intensive intervention at some point during study participation). The cross-over was also perceived by stakeholders as important for retaining participants assigned to the Traditional group; given that these participants had often already received treatments that were similar to Traditional therapist-guided self-management, these participants could look forward to a novel treatment upon completion of follow-up. Finally, the cross-over provided some pilot data on the feasibility of providing an in-home gaming intervention absent continued therapist support.

## **Treatment fidelity**

The following procedures promoted adherence to the protocol for the 13 therapists at 5 sites (and additional student trainees operating under their supervision):

- therapists received full-day in-person training by Drs. Gauthier and/or Borstad prior to treating the first participant
- whenever possible, therapists first shadowed another therapist who had demonstrated mastery, and then were directly observed by that therapist prior to independently treating participants
- therapists were provided with a treatment packet for each participant that included a checklist and all required study forms to ensure that no treatment components were missed
- sessions for all interventions were video-taped and randomly checked for adherence to the protocol by an independent rater
- retraining was required prior to treating additional participants if the independent rater identified protocol deviations
- therapists participated in a virtual asynchronous “brush up” training mid-way through the study.

## **Assessment**

Assessors were naïve to which study group participants had been assigned to. Assessments were videotaped for quality control. This served three purposes: 1) feedback to newly trained assessors on their performance, 2) to establish a sense of accountability to the standardized testing procedure, and 3) accurate measurement (e.g., testing footage could be re-examined in cases of unclear handwriting on assessment forms).

### **Primary Outcomes**

*Stakeholder input informed selection of outcome measures:* Qualitative analysis of feedback from our Advisory Board indicated two main therapy objectives: 1) regaining sufficient motor control to accomplish daily tasks/hobbies independently and 2) decreasing the time/effort required to perform tasks.

*Arm use:* The Motor Activity Log (MAL)^31,32^ assessed arm use for daily activities; each of 28 ADLs is self-rated on an 11-point scale from 0 (no use) to 5 (normal ability), assessed at 0·5 point intervals.

*Motor function/speed:* To address stakeholders’ priority for improving motor efficiency, the Wolf Motor Function Test (WMFT) measured the time to complete standardized functional movements (e.g., turning over cards, lifting a can to one’s mouth, folding a towel).^33-35^ WMFT performance time scores were natural log transformed prior to analysis, following precedent.^36^ The natural log transform renders positively skewed WMFT performance time data approximately normally distributed. A natural log scale can also better characterize clinical improvement because it approximates percentage change. For example, given equal 10 second performance time improvements, an improvement from 12 seconds to 2 seconds is more clinically meaningful than an improvement from 112 to 102 seconds; the natural log transformation accounts for this.

### **Secondary/exploratory outcomes:**

Secondary/exploratory outcomes for this trial are the Quality of Life in Neurological Disorders (Neuro-QoL)], 9-Hole Peg Test,^37^ Semmes‑Weinstein Touch Test monofilaments,^43-46^ and accelerometry. Sensation (Touch Test), cognition (Montreal Cognitive Assessment), and adherence (e.g., duration of game play) were examined for their impact on treatment response. Each measurement is described below.

The *Neuro-QoL*^38^ is a computerized adaptive test (CAT) that measures self-reported health-related quality of life for individuals with neurological disorders. It assesses aspects of physical, cognitive, emotional, and social functioning that are important to stakeholders and was developed using patient-centered methods.^38-40^ A CAT assessment was selected by the Advisory Board due to its significantly shorter administration time (about 4 minutes compared to 20 minutes for the Stroke-Specific Quality of Life assessment) and by researchers because it resulted from an NIH-funded initiative to develop common data elements for measuring quality of life in clinical studies. Stakeholders identified the Anxiety, Fatigue, Lower Extremity Mobility, Wellbeing, Sleep, Social Roles and Activities, and Cognition scales as most relevant to them. While the Upper Extremity scale also felt relevant to them, they felt that this was already captured effectively through the MAL. Each Neuro-QoL domain is reported as a T-score (mean of 50 and standard deviation of 10). The Neuro-QoL assessment was very new at the time that this research began, so it largely lacked psychometric validation. Recent work has provided some psychometric validation, for example moderate to strong cross-sectional correlations have been observed between the Neuro-QoL and the SF-36 quality of life assessments^41^ and the Neuro-QoL has demonstrated responsiveness to change on most scales amongst a population of individuals with Huntington's Disease.^42^ Other psychometric properties of the assessment appear weaker, however. A recent paper by Healy and colleagues^41^ showed 1) a compressed range of scores (standard deviations around 7) on this assessment, 2) that the CAT version of the test had “mild or no correlation with any clinical outcome measures including anxiety, depression, fatigue, and positive affect,” and 3) that change on the Neuro-QoL correlated only very weakly or not at all with change on the SF-36, another patient reported quality of life measure. The Neuro-QoL CAT is relatively new, so published accounts of its psychometric properties are limited^41,42^ and have yet to be established in a chronic stroke population. Results of analyses on this assessment should thus be interpreted cautiously.

The *Semmes‑Weinstein Touch Test* monofilament was included because stroke survivors identify loss of tactile sensation as a barrier to use of their more affected arm. Pilot work also showed a trend towards improved sensation amongst participants who were treated with the gaming self-management intervention.^12^ The monofilaments test quantifies the index finger’s threshold for detecting touch (in grams of pressure)^43^ with acceptable interrater^44^ and test-retest reliability.^45^ Touch test data was log transformed as recommended for analysis.^46^

The *9-hole Peg Test* was included in the assessment battery because it is commonly used to measure distal (fine-motor) improvement within clinical settings due to its short (2 minute) administration time. It is also the test of manual dexterity included in the NIH Toolbox.

*Adherence* to gaming self-management was measured via the gaming system. The gaming system logged both body movement and game actions. A multi-paradigm computational approach was employed post-hoc to automatically remove epochs of no movement and durations of artifact from the play time calculation (e.g., participant may answer the phone without pausing the system or begin exhibiting patterns of movement that are inconsistent with engagement in therapeutic game play).^47^ This computational approach also used features from the Microsoft Kinect skeleton (e.g., arm length, torso length) to remove any periods of time in which a different person was captured by the gaming system.^47^

The *Montreal Cognitive Assessment* (MoCA) is a rapid screening measure for detecting mild cognitive impairment with greater sensitivity than other commonly used screening tools (e.g., Mini-Mental State Examination).^48^ It has excellent specificity and excellent sensitivity for distinguishing participants with mild cognitive impairment and dementia from healthy controls.^49^ This measure was only collected at baseline to examine as a potential covariate.

*Accelerometry* quantifies the number of movements made by the paretic versus non-paretic upper extremities, similar to how a pedometer calculates number of steps. Smart-watches worn on both wrists (PebbleTime) measured tri-axial acceleration and counted the number of movements within each 24-hour period. A movement was logged when the sensor measured acceleration exceeding 2 g for at least 500 ms. Movement counts of the paretic upper extremity were reported relative to those of the less affected upper extremity to quantify proportional arm use. While accelerometry is an objective measure, it is notably less specific to functional activities than behavioral scales such as the MAL. Accelerometry captures both task-oriented and nonfunctional movements, as well as passive movements (the paretic arm being repositioned using the less affected arm). As most arm movements are not tasked oriented (e.g., gesturing with the hands while talking, habitual sensory stimulation such as twirling hair or smoothing clothing), accelerometry measures overall suppression of movement, rather than arm use for daily activities.

The *Brief Kinesthesia Test* quantifies error in targeted reaching to evaluate kinesthetic impairment. The evaluator moves the participant’s index finger to new locations on an 8·5” x 11” sheet of paper, then back to the original positions while vision is obscured. The participant then has to replicate the movement trajectories while vision remains obscured. The score on the measure is the summed distance in cm between each of the four targets and the endpoint of the participants’ movement. To our knowledge, the psychometric properties of the measure have yet to be established for individuals with post-stroke sensorimotor impairment.

## **Time frame for the study**

The Stroke Advisory Group provided feedback that a more compressed intervention is preferable to one that occurs over many months. They thus determined that a 3-week schedule would preserve the compressed pace of traditional CI therapy, while also allowing some flexibility in scheduling transportation and other commitments. A follow-up of 6 months was the longest that could be feasibly accomplished given the project budget period.

## **Procedures to maximize adherence and retention**

To maximize retention, all therapy and testing sessions were scheduled at the time of enrollment. A financial incentive of $50 per occasion was provided to encourage attendance at testing sessions. A thank-you letter was sent to participants a month after treatment completion with reminder of their follow-up testing date. Members of the Advisory Board suggested the following adherence-enhancing measures that were employed uniformly for this trial: 1) feedback regarding adherence during therapist encounters, 2) reminder phone-calls when necessary and feasible (to attend in-person therapy, complete scheduled at-home game play, and perform home exercises), 3) providing an instructional DVD/ video links for the participant and family (e.g., to educate/inform family members about CI therapy), and 4) giving participants a T-shirt at study completion if >90% adherence was achieved.

## **Analytical and statistical approaches:**

*Attrition:* Logistic regression examined whether study site, group, number of therapist encounters, or rural dwelling status influenced attrition. Each of the aforementioned factors was added in a forward stepwise procedure along with its interaction over time. A covariate was retained in the model if it significantly (*p* < .05) influenced overall attrition or attrition over time. Given the collinearity between group and number of therapist encounters, the two models (1. group and group by time and 2. number of therapist encounters and number of therapist encounters by time) were examined separately and the model with the lowest Bayesian Information Criterion was retained.

*Outlier detection:* Extreme outliers can skew overall treatment estimates. Extreme outliers on the MAL could indicate one or more of the following: 1) failure of the tester to follow the testing manual (i.e., paraphrased or incomplete instructions), 2) a participant misunderstanding the scale, 3) a participant’s limited awareness of his/her deficits, or 4) a participant’s poor memory of how the weaker arm was used during activities over the two days prior to MAL administration. Given that participants with significant cognitive impairments were included in the sample and that the MAL relies on accurate self-report, it was necessary to examine the data for outliers more than 3 standard deviations from the mean. Outliers were replaced following Random Forest Imputation (described in more detail in “Missing data” below). Outlier analysis was also conducted on the WMFT and Touch Test data to detect potential data entry errors, but no outliers were replaced.

Multiple Imputations of Missing data: Multiple imputation was performed by chained equations (MICE) in R.^50^ MICE employed 800 iterations^51^ of the random forest (RF) prediction algorithm to estimate the missing data from available data.^50^ RF is a machine learning technique that discovers nonlinear and complex relations between different variables in a dataset, thus providing a more accurate and less biased estimation of missing data compared to popular imputation techniques such as predictive mean matching or multiple regression^52-55^ that may be prone to overfitting and/or rely too heavily on particular participant features.^56,57^ RF utilized group assignment, clinical assessment data (summary scores on the touch test, MoCA, WMFT, and MAL, as well as individual item responses on the WMFT and MAL), and select demographic information (affected side, gender, handedness) to estimate the missing data. To verify that the imputed values were not dependent on RF’s initial randomly generated parameters (they were not), RF was repeated 5 times, each time with randomly generated initial RF parameters. No appreciable change in the estimates was realized as the number of iterations was further increased above 800, suggesting that 800 repetitions was sufficient.

Tests of Statistical Assumptions for Mixed Effect General Linear Model: Histograms and quantile-quantile plots were generated in Matlab for visual inspection. The Anderson-Darling test quantified whether residuals were normally distributed. Bartlett’s test quantified whether variances were equal across groups.

Intent-to-treat analysis: Strict intent-to-treat analysis was not possible because we lacked substantive data on participants who dropped out of the study before they received a pre-treatment assessment. As such, participants who began treatment were included in a modified intent-to-treat analysis. In the event of significant group x time interactions, two-tailed pairwise comparisons between the gaming groups (Self-Gaming and Tele-Gaming) and the two active comparators (CI therapy and Traditional therapist-guided self-management) were examined. The pairwise comparison between the two active comparators (CI therapy versus Traditional therapist-guided self-management) was not considered meaningful because the two interventions were not dose matched on most elements of the treatment (CI therapy employed more than double the number of therapist contacts and 7 times the amount of therapist time). The pairwise comparison between the two gaming groups on the sensory and motor outcomes was not considered especially meaningful because the two gaming treatments tested two different implementations of the flipped model of care and thus differed only minimally from one another (Tele-game had 6 additional brief teleconsultations with a therapist compared to Self-Gaming).

Analysis of Covariates: Each of the aforementioned covariates was added one at a time to the model. Covariates that have shown prior evidence of association were given precedence. For example, baseline Wolf Motor Function test score was added as the initial covariate for the Wolf Motor Function Test outcome given that prior work has shown proportional improvements in motor function (larger improvements on the outcome measure for those with initially poorer function). Prior to adding a new covariate, its multicollinearity was assessed via cross-correlation plots, examining its influence on the standard errors of the estimates for the group x time interaction, examining whether adding the additional predictor dramatically changed existing estimates in unexpected ways, and ensuring that the Variance Inflation Factor for the group x time effect did not exceed 5. Covariates exhibiting evidence of multicollinearity with the main effects of interest (group x time) were not added. A covariate that was collinear with another covariate, but not with the group x time interaction, could enter the model if the addition of the second covariate reduced the Bayesian Information Criterion. A covariate was added to the model if it significantly (*p* < .05) influenced treatment response or comparative treatment response without adversely influencing the Bayesian Information Criterion of the model. Three-way factor x time x group interactions were examined for factors that influenced treatment outcome (factor x time effect). Covariates and 3-way interactions were removed from the model when the addition of other covariates reduced their statistical significance (*p* > .10). To determine whether the observed relationships were linear, added variable regression plots^58^ displayed the added variable against the residual space of the other covariates.

## **Regulatory**

Regulatory oversight was conducted by the Institutional Review Boards (IRB) of The Ohio State University, OhioHealth, University of Alabama at Birmingham, and Missouri University. Providence Medford Medical Center IRB ceded review to The Ohio State University (central IRB).

# e-Results

## **Recruitment**

To maximize the external validity of the study, geographic, ethnic, and rural/urban diversity was emphasized through the choice of study sites. Recruitment methods also mainly targeted individuals in the community, rather than through particular hospital services. The most successful of these strategies was to recruit through direct mail. Prospective participants were identified by mining electronic medical records for ICD-9 and ICD-10 codes that indicated potential eligibility (e.g., diagnosis of stroke and hemiparesis). These potentially eligible individuals were then sent a direct mailing via United States Postal Service with an informational brochure and contact information of the study team. Two sites (UAB, Missouri) leveraged opt-out telephone registries of individuals that were treated for stroke and could be contacted for study participation. Use of social media, radio advertisements, and flyers at local libraries/community centers were also briefly tried with minimal impact. Recruitment continued until 6 months prior to termination of the study contract (to accommodate for the 6 month follow-up).

A participant was considered screened if a staff member had verbal or written reciprocal dialogue with an individual or family member about study participation. A participant was considered enrolled if they signed an informed consent form. The proportion of screened participants that ultimately enrolled in the study varied by site. The two sites that largely employed clinician referrals for recruitment had the highest enrollment rates (PMMC, OhioHealth), but relatively small overall enrollment given the limited reach of this approach. The direct mail recruitment strategy employed at OSU was successful at overall reach (exceeding target enrollment) as well as at targeting potentially eligible participants (40% of screened participants were enrolled). Telephone outreach (UAB, Missouri) was less successful. eTable 6 shows enrollment and reasons for exclusion by site.

### **eTable 6: Number screened and enrolled by site and reasons for exclusion.**

| *N*=1291 | **OSU** | **PMMC** | **OhioHealth** | **UAB** | **Missouri** |
| --- | --- | --- | --- | --- | --- |
| **Screened** | 251 | 20 | 38 | 444 | 538 |
| **Enrolled** | 101 (40%) | 10 (50%) | 17 (45%) | 55 (12%) | 10 (2%) |
| **Not interested** | 24 (10%) | 3 (15%) | 3 (8%) | 106 (24%) | 165 (31%) |
| **Minimal upper extremity impairment** | 35 (14%) | 0 (0%) | 13 (34%) | 82 (18%) | 106 (20%) |
| **Insufficient upper extremity ability** | 67 (27%) | 4 (20%) | 2 (5%) | 122 (27%) | 14 (3%) |
| **Deceased or medical contraindication** | 11 (4%) | 0 (0%) | 1 (3%) | 27 (6%) | 142 (26%) |
| **Receiving Botox in paretic extremity** | 5 (2%) | 0 (0%) | 2 (5%) | 2 (0%) | 5 (1%) |
| **Unable to travel** | 8 (3%) | 3 (15%) | 0 (0%) | 4 (1%) | 75 (14%) |
| **No diagnosis of stroke** | 0 (0%) | 0 (0%) | 0 (0%) | 32 (7%) | 18 (3%) |
| **Age <18** | 0 (0%) | 0 (0%) | 0 (0%) | 1 (0%) | 0 (0%) |
| **Received CI therapy previously** | 0 (0%) | 0 (0%) | 0 (0%) | 4 (1%) | 0 (0%) |
| **Incarcerated** | 0 (0%) | 0 (0%) | 0 (0%) | 0 (0%) | 3 (1%) |
| **Too acute** | 0 (0%) | 0 (0%) | 0 (0%) | 9 (2%) | 0 (0%) |

## **Participants**

One CI therapy participant was withdrawn during treatment after medical records revealed that an orthopedic injury caused the motor impairment. This participant was not included in the intent-to-treat analysis. Attrition during treatment was not significantly related to study group or to any measured participant factors (motor scores at baseline on WMFT and MAL, age, time in years since the stroke, Touch Test performance, MoCA score, whether or not the dominant hand was affected, or rural location, eTable 7). Attrition in follow-up was 24·7%, and disproportionately occurred in the two groups that had received the least amount of therapist contact during the treatment phase (36% vs 17%, *p* = .01) and at one study site (*p* < .01, eTable 7, eTable 8), while attrition for the other groups and sites was lower than in previous reports.^59^ As follow-up attrition appears to be related to group assignment, follow-up treatment change estimates and comparative treatment effects (particularly amongst the Self-Gaming and Traditional groups) should be interpreted with caution.

The study sample was disproportionately male (64% male, p=.001), reflective of the 41% higher stroke prevalence amongst males in the general population.^60^ Disproportionally more males were randomized to the Standard Care group and fewer to the gaming groups (Chi squared = 10·07, *p*=.02). Aside from the gender imbalance between groups, there were no other significant demographic differences between groups at baseline. Baseline characteristics of participants by study group are shown in Table 2 and by site in eTable 9.

## **Attrition**

**eTable 7. Characteristics of participants by attrition status.** Within-treatment attrition, follow-up attrition, and retention are quantified relative to the number of eligible participants who began treatment.

|  | **Pre-treatment attrition** *N*=193 | **Treatment attrition** *N*=167 | **Follow-up attrition** *N*=167 | **Retained**  *N*=167 |
| --- | --- | --- | --- | --- |
| **Total** | 25 (13%) | 17 (10%) | 37 (22%) | 113 (68%) |
| **Self-Gaming** | 4 (8%) | 6 (14%) | 13 (30%) ^a^ | 25 (57%) ^a^ |
| **Tele-Gaming** | 6 (12%) | 4 (9%) | 6 (13%) | 35 (78%) |
| **CI therapy** | 9 (18%) | 2 (5%) | 7 (17%) | 31 (78%) |
| **Standard Care** | 6 (14%) | 5 (13%) | 11 (29%) ^a^ | 22 (58%) ^a^ |
| **Male** | 13 (11%) | 11 (10%) | 26 (24%) | 73 (66%) |
| **Right Affected** | 15 (15%) | 8 (9%) | 17 (20%) | 61 (71%) |
| **Rural** | 11 (17%) | 9 (17%) | 9 (17%) | 34 (65%) |
| **Age** | 55 (13) | 58 (10) | 63 (14) | 59 (15) |
| **Chronicity** | 6 (7) | 4 (3) | 5 (6) | 5 (8) |
| **MoCA** | unknown | 21 (6) | 21 (6) | 22 (6) |
| **Touch Test** | unknown | -0·39 (2·72) | -0·23 (3·07) | -0·24 (2·61) |
| **Baseline WMFT** | unknown | 1·48 (0·90) | 1·99 (1·09) | 1·66 (0·95) |
| **Baseline MAL** | unknown | 1·5 (1·1) | 1·3 (0·7) | 1·5 (0·9) |
| Data are n (%), mean (SD).  ^a^ Significantly greater attrition at follow-up amongst the groups that had fewer therapist consultations | | | | |

### **eTable 8: Enrollment and attrition by site.**

|  | **Enrolled** | **Attrition pre-treatment** | **Attrition during treatment*** | **Attrition during follow-up** |
| --- | --- | --- | --- | --- |
| **Total** | 193 | 25 (13%) | 17 (9%) | 37 (19%) |
| **OSU** | 101 | 10 (10%) | 9 (9%) | 14 (14%) |
| **OhioHealth** | 17 | 0 (0%) | 0 (0%) | 2 (12%) |
| **UAB** ^a^ | 55 | 13 (24%) | 6 (11%) | 19 (35%) |
| **PMMC** | 10 | 0 (0%) | 1 (10%) | 1 (10%) |
| **Missouri** | 10 | 2 (20%) | 2 (20%) | 1 (10%) |
| ^a^ Significantly greater attrition  * Excludes the participant that was withdrawn for no stroke diagnosis | | | | |

**eTable 9. Baseline characteristics by study site.**

| *N*=193 | **OSU**  *n* = 101 | **OhioHealth**  *n* = 17 | **UAB**  *n* = 55 | **PMMC**  *n* = 10 | **Missouri**  *n* = 10 | ***p*** |
| --- | --- | --- | --- | --- | --- | --- |
| **% Male** | 59 | 76 | 65 | 70 | 70 | 0·66 |
| **% Right Affected** | 50 | 82 | 49 | 50 | 50 | 0·15 |
| **% Rural** (n=184) | 31 | 14 | 31 | 50 | 80 | 0·01 |
| **% Caucasian** | 66 | 71 | 54 | 80 | 80 | 0·01 |
| **% African American** | 28 | 6 | 43 | 0 | 0 |  |
| **% Asian** | 2 | 12 | 2 | 20 | 0 |  |
| **% Other race/ethnicity** | 1 | 0 | 0 | 0 | 0 |  |
| **% Mild cognitive impairment** | 41 | 35 | 52 | 30 | 22 | 0·42 ^a^ |
| **% Very poor cognition** | 14 | 18 | 5 | 10 | 33 |  |
| **% Diminished light touch** | 40 | 24 | 36 | 40 | 33 | 0·58 ^b^ |
| **% Less protective sensation** | 19 | 12 | 11 | 0 | 33 |  |
| **% Loss protective sensation** | 20 | 35 | 27 | 40 | 33 |  |
| **Age (years)** | 60 ± 16 | 67 ± 8 | 58 ± 15 | 59 ± 11 | 61 ± 10 | 0·18 |
| **Chronicity (years)** | 4·7 ± 6·7 | 2·5 ± 3·1 | 6·4 ± 10 | 5·7 ± 7·7 | 1·7 ± 1·3 | 0·27 |
| **MoCA** (*n*=172) | 21·5 ± 5·62 | 21·5 ± 5·7 | 22·6 ± 5 | 23·3 ± 7·4 | 18·9 ± 9·9 | 0·42 |
| **Baseline WMFT** | 1·7 ± 1·1 | 1·3 ± 0·9 | 1·9 ± 0·9 | 1·7 ± 0·8 | 1·5 ± 0·8 | 0·31 |
| **Baseline MAL** | 1·6 ± 0·9 | 1·7 ± 1 | 1·1 ± 0·7 | 1·4 ± 1 | 1·9 ± 1·1 | 0·03 |
| ^a^ *p*-value for ANOVA of MoCA scores  ^b^ *p*-value for ANOVA of log transformed Semmes Weinstein Monofilament Touch Test scores | | | | | | |

## **Adverse Events**

Twelve participants experienced medical events unrelated to the study; ten of whom discontinued participation as a result. Two participants reported study-related adverse events. One gaming participant experienced bruising from wearing the activity tracker too tightly and another experienced nausea secondary to muscle soreness. Neither adverse event affected study participation and both resolved quickly.

## **Missing data**

### **Primary Outcomes**

Primary outcomes data for both measures were captured for all but one participant who remained in the study. One UAB participant with transportation challenges only received the MAL at post-treatment due to inability to travel for testing (the MAL was administered via teleconference). This same participant arrived late to the follow-up testing session, so only the WMFT and Touch Test were administered (participant could not be contacted afterwards to complete the MAL remotely).

### **Exploratory Outcomes**

*Nine-Hole Peg Test:* There were 7 participants with missing data on the Nine Hole Peg Test at post-treatment and two participants with missing data at follow-up.

*Touch test monofilaments:* There were 6 participants with missing data on the Touch Test at post-treatment and follow-up. Five instances of missing Touch Test data were due to testing protocol deviation at one of the sites; the other instance was due to excessive hypotonicity that precluded opening the participants’ hand to administer the test.

*Neuro-QoL:* Data was missing for 21 participants at pre-treatment, 16 participants at post-treatment, and 33 participants at follow-up. Missing Neuro-QoL data resulted from hospital firewall or other issues accessing the online computerized adaptive assessment.

*Brief Kinesthesia Test:* There were 31 participants with missing data due to insufficient motor ability to perform the test. One additional participant was missing data at post-treatment and follow-up due to arriving late to the assessment.

*Accelerometry:* Reliable accelerometry data was available for just 77 participants. Accelerometers were unavailable (on order) for the first 19 participants enrolled into the trial. The remainder of missing data was primarily due to participants forgetting to charge the devices, forgetting to don the devices, donning the devices for an insufficient amount of time, temporarily misplacing the devices, or only gathering data from one wrist at any given time (such that the affected versus less affected arm use ratio could not be calculated). Some missing data also resulted from errors committed by the research assistants, such as resetting a device prior to downloading the data or accidentally downloading the data a second time from the same device (e.g., that worn on the less affect arm) and mistakenly attributing this data to the other arm (e.g., the more affected arm). To improve compliance, future research could employ devices that are smaller and that provide relevant feedback to the wearer (e.g., monitor sleep quality or general activity) so that they are better tolerated by participants, could provide daily reminders to don the devices, and could employ waterproof devices with >30 days of battery life to avoid the need to don/doff the sensors.

## **Post-hoc power calculation**

Post-hoc power analysis is informative for two reasons: 1) our initial power estimates were derived from a previous study with a much smaller sample size and more restrictive inclusion criteria than what was employed in the present study and thus may not fully generalize to the current study; 2) the desired sample size was not met, so it is helpful to estimate the potential for Type II error within the results. Post-hoc power was estimated for each primary outcome using a Monte Carlo approach in MATLAB with the following parameters: 5000 permutations, α = .05, and sample sizes from the study sample. For each permutation, simulated data was generated using the within-group variability from the present study and a comparative treatment effect equal to the MCID between one study group and the other 3 groups. For example, the MCID for the MAL is 1 point, so data was simulated such that one group showed a mean improvement that was 1 point greater than the other three groups, whose mean improvements did not differ from one another in the simulation. As the MCID for the WMFT is proportional to the initial performance time (16% reduction), the magnitude of the WMFT MCID was calculated using baseline WMFT performance time data from the current study.

## **Outlier adjustment**

Three pre-treatment datapoints on the MAL were flagged as outliers and imputed via RFI. Two had excessively high scores (*z* > 3) that were inconsistent with MAL scores obtained during screening. The third approached the threshold for outlier detection (*z* > 2) and video review revealed that instructions had been read incorrectly to the participant during the pre-treatment MAL administration. One outlier in follow-up was adjusted via RFI; this participant had very poor cognition (MoCA = 6) and his report of fair-good quality of arm use during follow-up differed from his clinical presentation. A sensitivity analysis revealed that imputing these outliers did not change the final treatment model, but did predictably reduce the standard errors of the estimates. One participant with cognitive impairment was removed altogether from the MAL analysis (list-wise deletion in lieu of interpolating MAL data from all 3 time points) due to inconsistent patterns of responding (e.g., different items rated as not attempted at pre-treatment versus post-treatment versus follow-up, better quality ratings for some harder items than for easier items.^31^ For the WMFT, one CI therapy participant showed unusually strong recovery during the treatment period (*z* < -3) and one Standard Care participant showed exceptionally strong recovery during the 6-month follow-up period (*z* < -3); video review confirmed accurate test administration and scoring in both of these cases, so the data was left unaltered.

## **Tests of Normality**

Tests of normality revealed a slight right “heavy tail” for both primary outcome measures. Permutation analysis was thus utilized to generate a null distribution from the study data to determine the impact of this minor violation of the normality assumption on the probability values generated by the model; group assignment was randomly shuffled over 5000 iterations and the probability value for the group x time interaction was stored with each iteration. This procedure revealed minimal impact of violating the normality assumption on the probability values generated by the model. Simulation studies (e.g., Schielzeth, 2020) similarly show that linear mixed-effect models are robust to skewed data. Bartlett’s test revealed that variances did not differ significantly between the groups.

##

## **Supplemental MAL results**

### **eTable 10: General linear mixed effects model for the MAL Model ^a^**

| **Term** | **Explanation** |
| --- | --- |
| mal | Outcome measure |
| 1 | intercept |
| group*time | main effects of group and time and their interaction |
| wmftpre:time | fixed effect of baseline motor function on change over time |
| malpre:time | fixed effect of baseline daily arm use on change over time |
| (1+time\|subjs) | random intercepts and random slopes attributed to each participant |
| (1\|site) | random intercepts attributed to the 5 sites |
| ^a^ Full model: mal = 1 + group*time + wmftpre:time + malpre:time + (1+time\|subjs) + (1\|site) | |

**eTable 11. Effect sizes for the patient characteristics associated with MAL response.** Effect sizes are expressed as partial slopes, meaning the extent to which a 1-point increase in baseline MAL and WMFT scores have on the MAL treatment change from baseline, adjusted for covariates in the mixed effects models.

|  | **Effect size (95% CI)** | ***p*** |
| --- | --- | --- |
| **Baseline MAL** | | |
| **treatment** | -0·20 (-0·29, -0·10) | <0·001 |
| **follow-up** | -0·39 (-0·53, -0·26) | <0·001 |
| **Baseline WMFT** | | |
| **treatment** | -0·19 (-0·29, -0·09) | <0·001 |
| **follow-up** | -0·11 (-0·25, 0·02) | 0·10 |

**eFigure 2: Gains in arm use versus baseline motor ability.** Poorer motor function at baseline was weakly associated with poorer improvement and retention of gains in daily arm use. The y-axis displays the residuals from the mixed effects general linear model after accounting for the fixed effects/interactions of group, time, baseline daily arm use, and the random intercept of study site. WMFT performance time scores were natural log transformed.


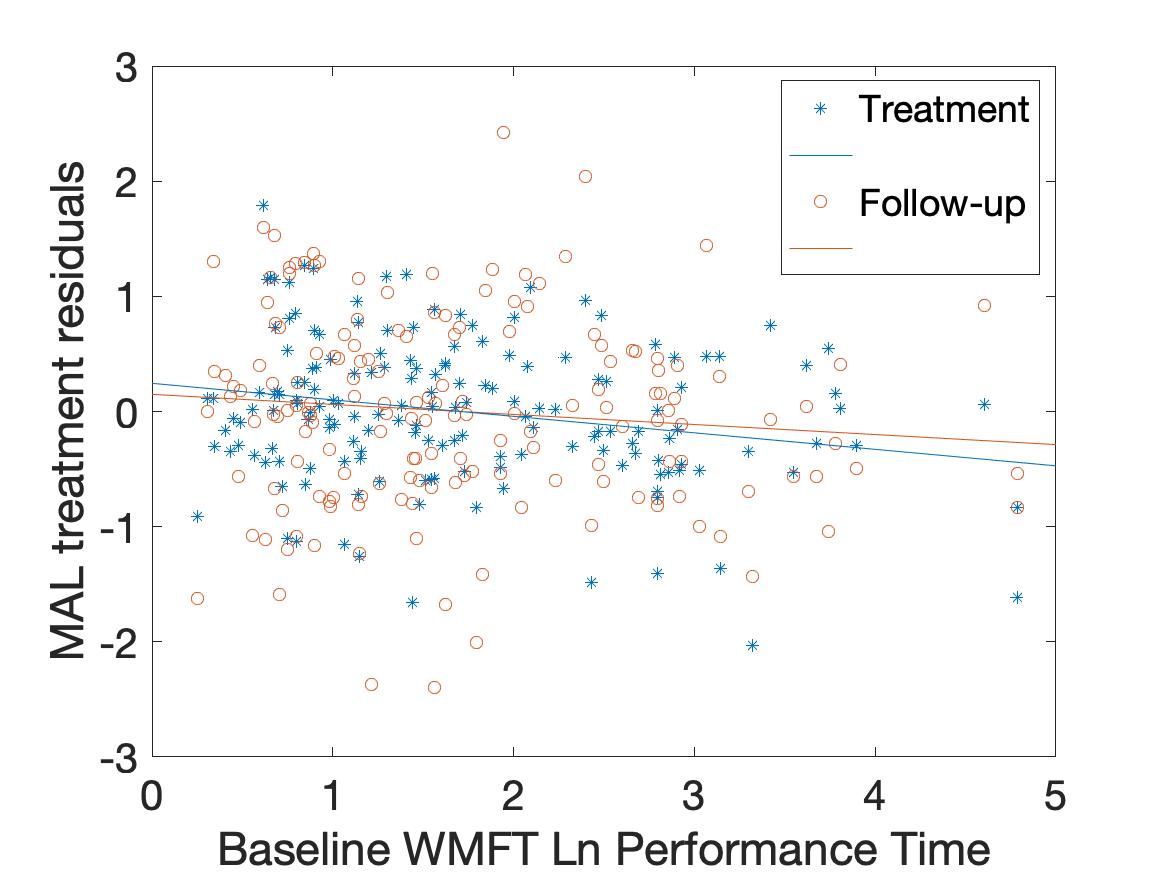


**eFigure 3: Gains in arm use versus baseline arm use.** Higher baseline MAL scores predicted smaller treatment-induced improvements on the MAL. The y-axis displays the residuals from the mixed effects general linear model after accounting for the random intercept of study site and the fixed effects of group, baseline motor ability, and gender.


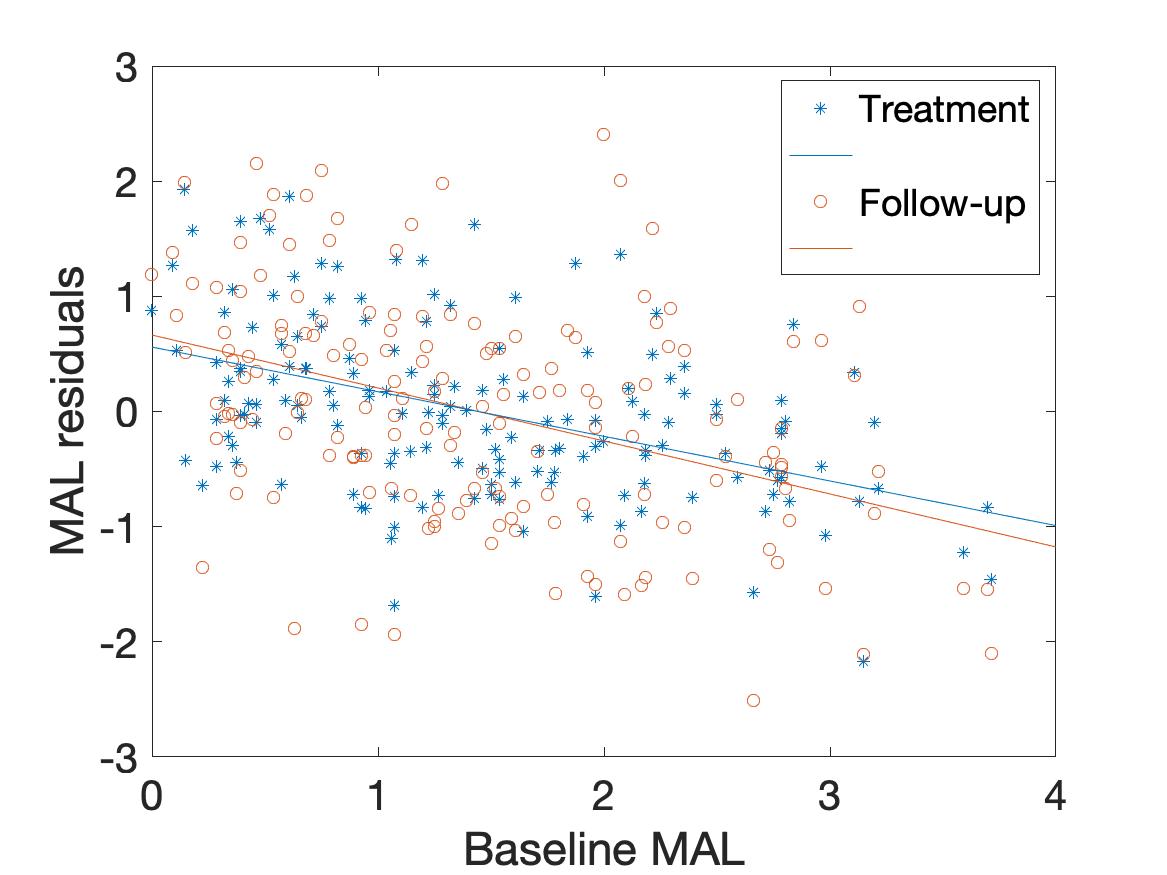


## **Supplemental WMFT Results**

**eFigure 4: Boxplot of proportional improvements by group on the WMFT.** Proportional improvement reflects the extent of improvement relative to the participant’s maximum possible improvement [(post-treatment – pre-treatment)/(best possible score – pre-treatment)]. The central marks are the medians, the edges of the box are the 25th and 75th percentiles, the whiskers extend to the most extreme datapoints that aren’t outliers (within 1·5 times the interquartile range from the outer limits of the interquartile range), and “+” indicates outliers.


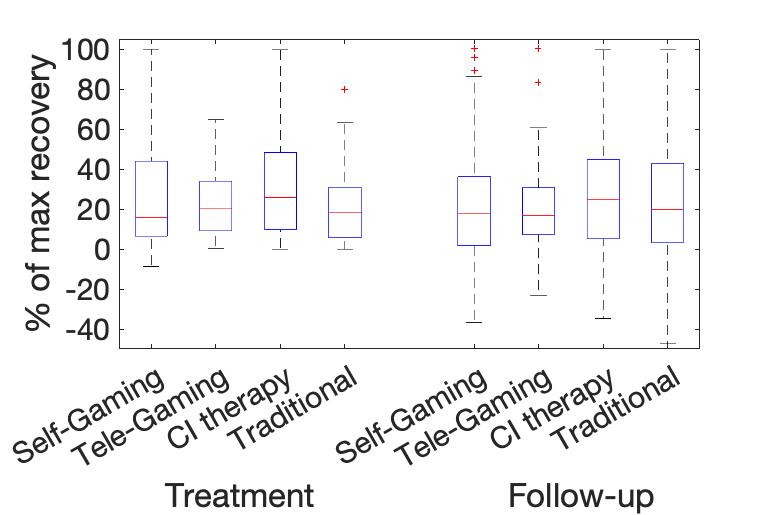


**eTable 12: General linear mixed effects model for the WMFT primary outcome:**

wmft = 1 + group*time + wmftpre:time + (1+time|subjs) + (1|site)

| **Term** | **Explanation** |
| --- | --- |
| wmft | Wolf Motor Function test primary outcome measure |
| 1 | intercept |
| group*time | main effects of group and time and their interaction. The interaction is the comparative treatment effect of interest. |
| wmftpre:time | fixed effects of baseline motor function on change over time |
| (1+time\|subjs) | random intercepts and random slopes attributed to each participant |
| (1\|site) | random intercepts attributed to the 5 sites |

**eTable 13· Effect sizes for the patient characteristics associated with WMFT response.** Effect sizes are expressed as partial slopes, meaning the effect that a 1-point increase in baseline WMFT scores has on the WMFT treatment change from baseline, adjusted for covariates in the mixed effects models.

|  | **Effect size (95% CI)** | ***p*** |
| --- | --- | --- |
| **Baseline WMFT** |  |  |
| **treatment** | 0·19 (0·14,0·24) | <.001 |
| **follow-up** | 0·53 (0·46, 0·60) | <.001 |

**eFigure 5: Gains in motor ability versus baseline motor ability.** Participants with poorer initial motor ability (higher scores) made more robust improvements in motor function (WMFT) that were retained through 6-month follow-up. The y-axis displays the residuals from the mixed effects general linear model after accounting for the fixed effects/interactions of group, time, and the random intercept of study site. WMFT performance time scores were natural log transformed.


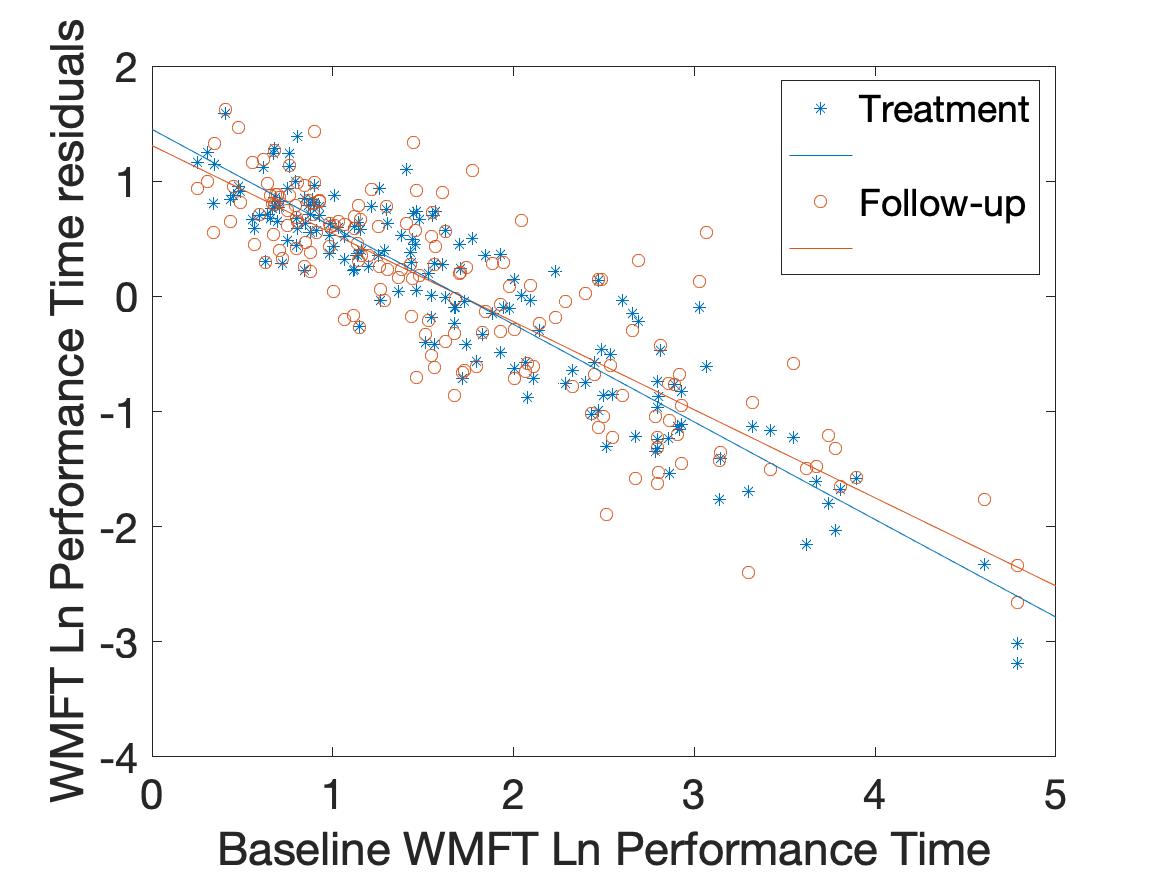


## **Adherence**

Adherence to self-management was incomplete. Adherence appeared to fluctuate with extent of therapist contact, with Tele-Gaming participants who had greater frequency of therapist contact showing a median adherence that was significantly better (almost double) that of the Self-Gaming group (eFigure 6).

Adherence to in-person rehabilitation was complete for the majority of participants. Four CI therapy participants missed between 2 and 10 hours of treatment due to illness or tardiness. One Traditional participant missed 1·75 hours of treatment due to tardiness and not showing up for an appointment. One Tele-Gaming participant no-showed to 1 hour of in-person treatment and to 1 brief teleconference.

Adherence was collinear with treatment group, so adherence could not be examined together with treatment group in the general linear model. When hours of motor practice (time spent gaming or on therapist-led motor practice) was included in the model instead of group, its interaction with time was not significant, suggesting the absence of a linear relationship between extent of motor practice and improvements in motor speed. Similarly, there was no evidence of a direct linear dose-response relationship within the subset of participants that received game-based motor intervention.

Kinematic data obtained from the gaming system throughout the course of gaming practice hints as to why a cross-sectional investigation of dose-response reveals no consistent relationship. There were clear negative exponential dose-response curves for most treatment responders, but they varied markedly between individuals, suggesting that dose-response is heterogeneous between participants. Kinematic dose-response data will be presented further in a separate publication.

A per protocol analysis examined the WMFT outcome for only those participants who completed the entire treatment as prescribed. As with the intent-to-treat analysis, no statistically significant or clinically meaningful between-group differences were observed (eFigure 7).

**eFigure 6: Boxplot of adherence to game play amongst the Tele-Gaming (right) and Self-Gaming (left) participants.** The number of hours spent engaging in active game play was computationally extracted from the gaming system logs.^47^ The box reflects the interquartile range (middle 50%) and the horizontal line within the box reflects the median. The whiskers reflect the most extreme values within the data. Tele-Gaming participants received identical gaming and in-clinic interventions to the Self-Gaming participants, with an additional 6 video consultation sessions (4 versus 10 therapist contacts). These six brief video consultations boosted median adherence to self-managed motor practice by 5·1 hours.


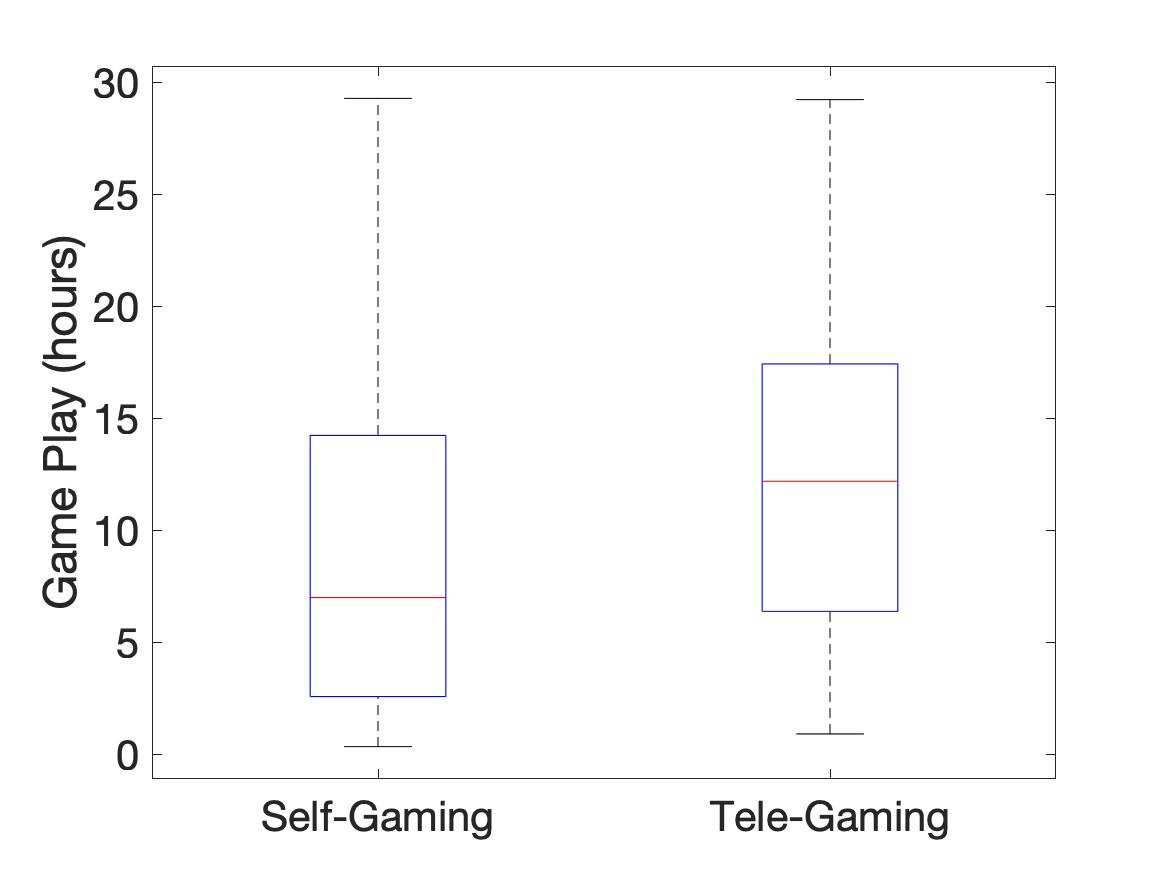


**eFigure 7: Per-protocol analysis of the WMFT** examined treatment change (natural log transformed) by group during the intervention period (blue, left) and 6-month follow-up (orange, right) amongst individuals who fully adhered to the motor practice. The possible range of the natural log transformed WMFT treatment change is -4·78 to 4·78, with a negative treatment change indicating improvement. Given log transformation, WMFT treatment changes approximate (but slightly overestimate) percent improvement, e.g., a difference of 0·1 log units is roughly equal to 10%. Consistent with the intent-to-treat analysis, comparative treatment effects were absent.


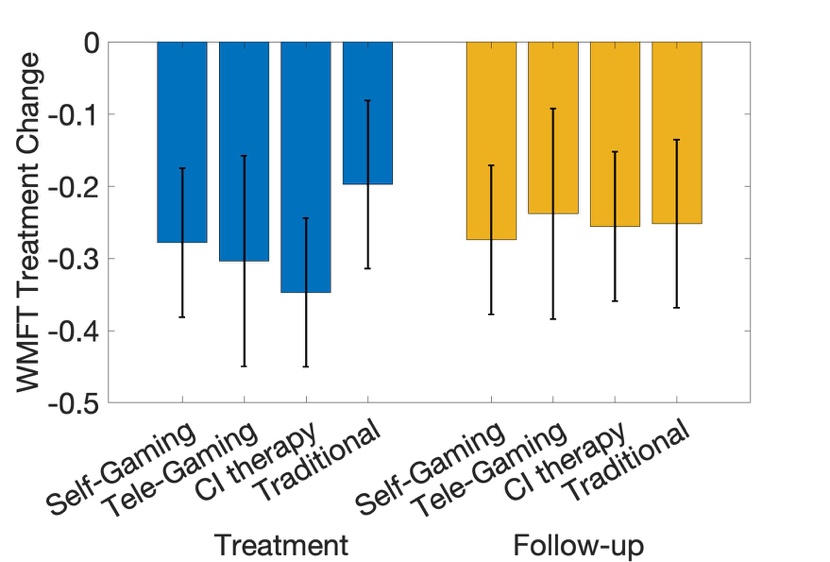


## **Traditional group crossed-over to Gaming self-management.**

The Traditional group was given the opportunity to receive in-home game play after completing the 6-month follow-up. Prior to taking the game home, this group received a 2-hour therapist consultation session in which they learned how to operate the game and were introduced to the behavioral components of the intervention, which they subsequently completed independently. The structure of this 2-hour session was identical to the first in-person consultation from the Self-Gaming and Tele-Gaming groups, as was the prescribed game play and agreed upon in-home task practice. Following this initial consultation, cross-over participants completed the remainder of the program independently (i.e., with no additional therapist consultation). While controlled comparisons cannot be drawn from this cross-over period, it provides some preliminary evidence of 1) the impact of therapist consultations on adherence to game play, 2) the impact of therapist consultations on improvements in daily arm use, and 3) whether a second bout of motor practice months later may continue to improve motor function.

Nineteen participants completed both the cross-over intervention and post-cross-over motor testing. Mixed-effect general linear models applied to these 19 complete cases examined the main effect of time on the primary outcome variables. Interaction terms were not included in the model given the small sample size.

Adherence was similar to the Self-Gaming group, with a median active game play time of 7 hours. Only one individual completed the prescribed 15 hours. Motor function continued to improve through the post-cross-over period. By the end of the cross-over period, participants attained mean motor gains of -0·34 (95% CI -0·61 to -0·07, eFigure 8, left). Sixty-eight percent of participants had achieved a clinically meaningful change in motor function by the end of the cross-over period. Statistically significant improvements in daily arm use were also achieved post-cross-over (0·84, 95%CI 0·51 to 1·17, eFigure 8, right), but these improvements were clinically meaningful for only 37% of individuals.

Improvements in daily arm use during the cross-over period were small, equal to one fifth of the MCID (Post-cross-over MAL – Follow-up MAL = 0·2). Even though participants were provided with the same behavioral treatment materials employed by the Self-Gaming and Tele-Gaming groups (all study forms used by the therapists, instruction from a therapist on how to employ the behavioral techniques, a computerized self-assessment of arm use to complete daily, and a video explaining how to apply the behavioral techniques), the majority failed to improve daily use of their paretic arm. This is in stark contrast to the gains in daily arm use achieved by the Self-Gaming and Tele-Gaming participants during the period of active intervention (1·3 and 1·5, respectively). The disparity suggests that interactive dialogue and accountability with a therapist is a driving force behind behavior change and that behavioral change cannot be effectively self-managed.

**eFigure 8: Continued improvement after 6-month cross-over to gaming.** WMFT scores at each timepoint are shown on the left. Performance time was natural log transformed. MAL scores at each timepoint are shown on the right. Error bars reflect 95% confidence intervals. Performance improved during each bout of active treatment and was maintained in the interval between treatments.


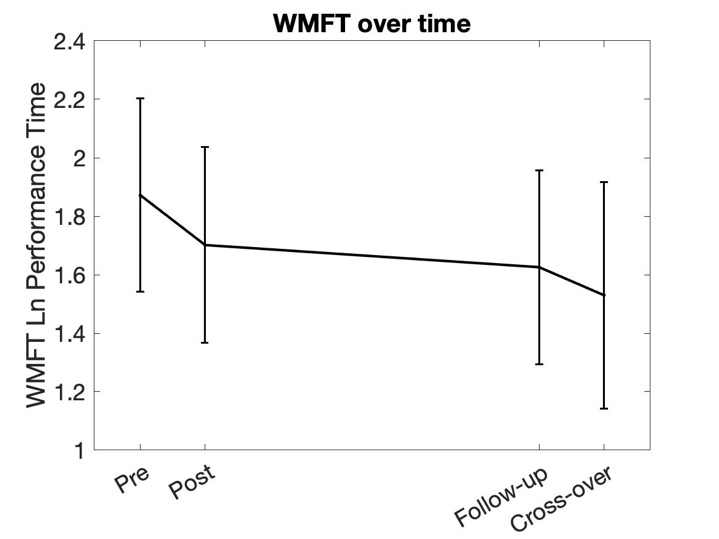

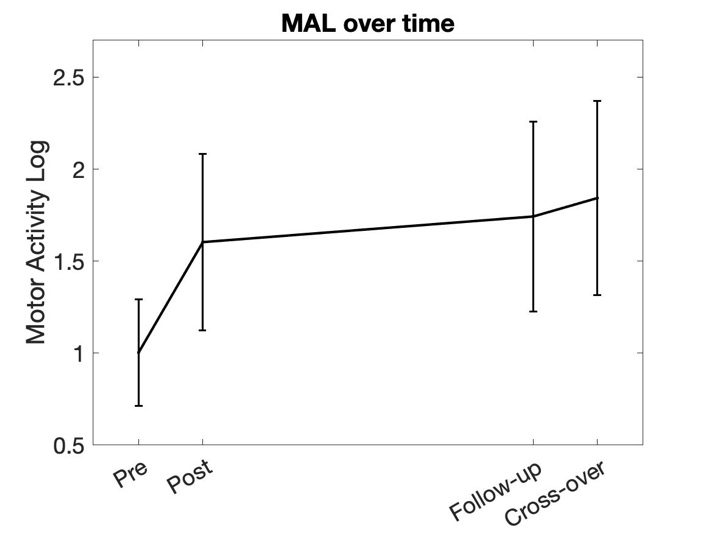


## **Analysis of Secondary Outcomes**

*Nine-Hole Peg Test:* Consistent with another stroke study,^61^ floor effects were present on the Nine-Hole Peg Test. This measure may thus underestimate the actual impact of treatment on distal motor function in the study sample. The majority (58%) of participants were unable to place all 9 pegs at baseline within 120 seconds. Sixty-three participants (38%) were unable to place any pegs at baseline and 23% were unable to place any pegs at any of the testing sessions. Nine Hole Peg data was converted to a rate metric for analysis, such that participants who were unable to place all 9 pegs could be included. The rate metric was expressed as the number of pegs that could be placed within a minute and was calculated thus: number of pegs placed*60 (s/min) / performance time (s). There was no meaningful improvement in Nine Hole Peg performance over time for any study group (median changes over time were about zero) and there were no between-group differences. This suggests that improvements to distal motor function several years post-stroke may be limited and/or require much larger therapy doses than those employed in the current study.

*Touch test monofilaments:* Touch sensation improved across all groups. Results will be published separately.

*Neuro-QoL:* There were no significant treatment-related improvements on the Neuro-QoL (main effect of time) and the mean treatment changes on all scales were smaller than the minimally detectable change for the measure.^62^ There were no significant comparative treatment effects (group by time interactions). These results are consistent with some prior work that shows that quality of life improvements from CI therapy are limited to activities of daily living and motor domains (i.e., did not generalize to the social, family, cognitive, mood domains, etc. that were measured here)^63^ or that general quality of life improvements are not generally realized from CI therapy.^64^ However, these findings are inconsistent with another previous study that showed quality of life improvements following CI therapy across multiple non-motor domains that strongly correlated with improvements in daily arm use.^65^ The inconsistencies in findings may be partially explained by differences in the assessments that were used, participant characteristics,^66^ or suboptimal psychometric properties of existing quality of life assessments.^41^ For example, in our sample, the composite T-score had a standard deviation of just 2·4 (standard deviation for T-scores should be 10) and each subscale had substantially lower than expected standard deviations, producing a restricted range for analysis). In sum, Neuro-QoL results should be interpreted with caution given inconsistently demonstrated validity of the assessment and the salient impact that mood and educational level have on self-reports of quality of life.^66^

*Brief Kinesthesia Test*: The Brief Kinesthesia Test is an exploratory measure of kinesthetic sense that does not yet have established reliability and validity in stroke. It exhibited unacceptable reliability in our sample. Scores at different time points were minimally correlated with scores at the other time points (*r*s ranged from 0·17 to 0·31). In the context of minimal overall improvement on the measure (7% mean improvement from baseline to post-treatment and 10% mean improvement from baseline to follow-up), one would expect scores from a reliable measure to remain consistent across time (i.e., highly correlated over time). Yet, marked score deviations across time were observed, reflective of inconsistent performance for the majority of participants. Results for the BKT were thus not analyzed further given concerns regarding its reliability, and thus its validity, for the stroke population.

*Accelerometry:* The ratio of the number of movements of the paretic versus less affected arm was highly consistent across time for each participant (test-retest reliability > 0·8 between recordings obtained on consecutive days, most frequently > 0·9). The paretic arm was used significantly less than the less affected arm (median = 45% as often). Frequency of movement did not change significantly as a result of treatment nor were any comparative treatment effects observed between groups. This suggests that while participants were able to successfully incorporate use of their paretic arm into specific and relevant daily tasks following behavioral treatment (as measured via the Motor Activity Log), they continued to exhibit an overall suppression of automatic movement (e.g., gestured only with the less affected arm during speech).

# Supplemental Statistical Analysis

The following subsections reflect additional analyses performed on the data with different ways of accounting for drop-out. The pattern of results using these alternate methods for replacing data were similar to the results presented in the main paper (Table 3).

## **Intent-to-treat analysis as originally planned in the published study protocol**

This analysis uses the last observation carried forward method to impute data lost to voluntary attrition, which essentially treats dropouts as complete non-responders. Random Forests Imputation (RFI) was still used to impute data for individuals who had to withdraw for medical reasons and for those lost to follow-up. The difference between this analysis and that presented in the main paper is that this analysis uses different methods of imputation depending on a person’s reason for dropping out of the study, whereas the main analysis uses RFI imputation for all missing data. This analysis will underestimate treatment effects given that it conservatively assigns no treatment change to voluntary dropouts, even though these individuals may have benefitted from the portion of the intervention that they had received.

**eTable 14. Descriptive Statistics of Supplemental Intent-to-treat analysis.** Means and standard deviations of each treatment group at each time-point. MAL scores are expressed as mean MAL ratings. WMFT scores are expressed as the mean of the natural log of performance times for each item and negative changes reflect improvement.

|  | | **Self-Gaming** | **Tele-Gaming** | **CI therapy** | **Traditional** |
| --- | --- | --- | --- | --- | --- |
| **MAL** | Pre-treatment | 1·5 ± 0·8 | 1·5 ± 0·9 | 1·5 ± 1·0 | 1·2 ± 0·8 |
|  | Post-treatment | 2·6 ± 0·9 | 3·1 ± 0·9 | 3·2 ± 1·0 | 1·7 ± 1·1 |
|  | Follow-up | 2·0 ± 1·0 | 2·4 ± 1·2 | 2·7 ± 1·2 | 1·6 ± 1·1 |
|  | Treatment change | 1·1 ± 0·8 | 1·5 ± 0·7 | 1·7 ± 0·7 | 0·5 ± 0·6 |
|  | Pre-tx to f/u change | 0·5 ± 0·8 | 0·9 ± 0·8 | 1·1 ± 0·8 | 0·4 ± 0·9 |
| **WMFT** | Pre-treatment | 1·64 ± 1·05 | 1·60 ± 0·91 | 1·82 ± 1·07 | 1·81 ± 0·92 |
|  | Post-treatment | 1·45 ± 1·00 | 1·32 ± 0·79 | 1·44 ± 0·97 | 1·64 ± 0·95 |
|  | Follow-up | 1·48 ± 0·95 | 1·38 ± 0·80 | 1·50 ± 0·98 | 1·58 ± 0·93 |
|  | Treatment change | -0·19 ± 0·30 | -0·28 ± 0·33 | -0·39 ± 0·35 | -0·17 ± 0·33 |
|  | Pre-tx to f/u change | -0·16 ± 0·42 | -0·22 ± 0·38 | -0·32 ± 0·52 | -0·23 ± 0·57 |

**eTable 15: Comparative treatment effects for Supplemental Intent-to-treat analysis.** Effect sizes reflect between-group pairwise comparisons adjusted for covariates in the final mixed effects general linear model (95% confidence interval). Rows labeled “treatment” and “6-month” show the post-treatment and follow-up scores relative to pre-treatment scores, respectively. A positive between-group difference for the MAL means that the group listed first in the comparison showed greater gains in arm use. A negative between-group difference for the WMFT means that the group listed first in the comparison showed greater gains.

|  | **Tele-Gaming vs**  **CI therapy** | **Self-Gaming vs**  **CI therapy** | **Tele-Gaming vs Traditional** | **Self-Gaming vs Traditional** |
| --- | --- | --- | --- | --- |
| **MAL treatment** | -0·2 (-0·4, 0·1) | -0·5 (-0·8, -0·3) ^s^ | 0·9 (0·6, 1·2) ^s^ | 0·5 (0·2, 0·8) ^s^ |
| **MAL 6-month** | -0·3 (-0·6, 0·0) | -0·6 (-0·8, -0·3) ^s^ | 0·4 (0·1, 0·8) ^s^ | 0·1 (-0·3, 0·4) |
| **WMFT treatment** | 0·11 (-0·03, 0·24) | 0·20 (0·06, 0·33) ^s^ | -0·10 (-0·24, 0·04) | -0·01 (-0·15, 0·13) |
| **WMFT 6-month** | 0·10 (-0·10, 0·30) | 0·16 (-0·04, 0·36) | 0·01 (-0·19, 0·21) | 0·07 (-0·13, 0·27) |
| ^s^ Statistically significant differences between groups | | | | |

###

## **Analysis of only existing data, no imputation performed.**

Descriptive statistics were compared to validate that the imputation of missing data did not dramatically change the treatment effects. This analysis used only the available data; no missing data was interpolated. Treatment effects were consistent with the intent-to-treat analyses reported in the main paper.

**eTable 16. Analysis without imputation.** Means and standard deviations of each treatment group at each time-point are shown. MAL scores are expressed as mean MAL ratings. WMFT scores are expressed as the mean of the natural log of performance times for each item. Negative WMFT changes reflect improvement.

|  | | **Self-Gaming** | **Tele-Gaming** | **CI therapy** | **Traditional** |
| --- | --- | --- | --- | --- | --- |
| **MAL**  *n*=166 | Pre-treatment | 1·5 ± 0·8 | 1·5 ± 0·9 | 1·5 ± 1·0 | 1·2 ± 0·8 |
|  | Post-treatment | 2·7 ± 0·9 | 3·1 ± 0·8 | 3·2 ± 1·0 | 1·8 ± 1·1 |
|  | Follow-up | 2·2 ± 1·1 | 2·4 ± 1·2 | 2·8 ± 1·1 | 1·8 ± 1·2 |
|  | Treatment change | 1·3 ± 0·7 | 1·6 ± 0·6 | 1·7 ± 0·7 | 0·6 ± 0·6 |
|  | Pre-tx to f/u change | 0·5 ± 0·9 | 0·9 ± 0·8 | 1·2 ± 0·8 | 0·6 ± 0·7 |
| **WMFT**  *n*=167 | Pre-treatment | 1·64 ± 1·05 | 1·60 ± 0·91 | 1·82 ± 1·07 | 1·81 ± 0·92 |
|  | Post-treatment | 1·49 ± 1·06 | 1·33 ± 0·74 | 1·42 ± 0·96 | 1·62 ± 0·97 |
|  | Follow-up | 1·45 ± 0·98 | 1·39 ± 0·79 | 1·29 ± 0·86 | 1·50 ± 0·77 |
|  | Treatment change | -0·22 ± 0·32 | -0·30 ± 0·33 | -0·39 ± 0·35 | -0·19 ± 0·33 |
|  | Pre-tx to f/u change | -0·14 ± 0·41 | -0·26 ± 0·38 | -0·36 ± 0·50 | -0·23 ± 0·61 |

# Limitations

## **Limitations to external validity:**

*Study sample*: While this study employed broader inclusion criteria (e.g., comorbidities, substantial cognitive impairment, etc.) than prior CI therapy studies,^4,67,68^ these findings can only be generalized to individuals who have some movement in both the proximal and distal upper extremity and who present with non-use (baseline MAL < 2·5). Differential recruitment across study sites also led to an over-representation of participants from a single site (52%) and geographic region (61%). Given the randomized design, the study population was also restricted to individuals who were willing and able to travel to in-person therapy and testing sessions (e.g., the proportion of rural individuals enrolled in the study was less than anticipated). A person’s geographical proximity and access (e.g., reliable transportation) plays a large role in determining their preferred balance of in-clinic versus self-management interventions. Thus, those who favor in-clinic treatments were likely over-represented in the study sample. This has the potential to introduce placebo-expectancy effects that could theoretically skew the comparative treatment effect in favor of therapist-led motor practice, i.e., towards the two comparator groups.

*Traditional comparator*: To maximize internal validity for the study, it was necessary to standardize Traditional therapist-guided self-management. The Traditional approach was informed by experienced occupational and physical therapists working in outpatient neurorehabilitation clinics within large academic medical centers. It thus may not be wholly representative of (and potentially better than) the treatment that stroke survivors receive in smaller rural clinics or through home health services that do not specialize in neurologic rehabilitation. This factor also represents a bias that works against the study hypotheses. This arm also did not employ modalities or devices that are sometimes, albeit infrequently, used in clinical settings to treat this population (e.g., functional electrical stimulation, robotic therapy, aquatic therapy).

*Missing data*: Data for some secondary outcomes was missing disproportionately from sites that were not located in Columbus Ohio, so the results of secondary outcomes may have more limited external validity.

## **Limitations to internal validity:**

*Design*: This study was designed to determine how a largely self-administered therapist-as-consultant treatment approach that emphasizes behavioral change differs in terms of practical effectiveness from therapist-administered in-clinic CI therapy and traditional therapist-guided self-management that emphasizes motor training. As such, the design does not allow for a direct comparison between gaming versus in-clinic treatment modalities because adherence in the gaming groups was imperfect (as expected) and the pace of treatment differed between gaming and CI therapy groups (e.g., breaks were administered to CI therapy participants between trials, whereas gaming therapy involved continuous high-repetition movement). Rather, the study captured what appears to be a negative feature of Self-Gaming and Traditional therapist-guided self-management, relatively low adherence to the motor training amongst some participants, which resulted from minimal direct therapist oversight in the gaming self-management groups.

*Measurement:* The MAL measures self-reported arm use for common daily activities. As such, improvements on the MAL may capture greater use of the arm for particular common daily activities without accompanying improvements in motor control. In other words, improvements on the MAL cannot be necessarily interpreted as improvements in the underlying coordination problems. Nonetheless, the MAL is a very useful outcome measure because therapies that lessen motor impairment with minimal impact on everyday arm use fail to affect a person’s daily functioning in a meaningful way.

As the MAL also depends on self-report, it is possible that its reliability may be affected by cognitive impairment, which is common post-stroke. Indeed, there was a 19% increase in variance of the MAL treatment effects as well as a few outlier MAL measurements amongst those with cognitive impairment. Despite this, post-hoc power to detect meaningful comparative treatment effects on the MAL was still greater than 99% and extent of cognitive impairment did not bias MAL comparative treatment effects. This suggests that the inclusion of individuals with cognitive impairment within the study sample may have slightly reduced the precision of estimates, yet also markedly improved its external validity.

The WMFT is a timed measure, so performance can improve in the absence of improvements to motor coordination. This trial lacks a well-studied measure that can document improvements in motor coordination specifically. The choice of outcome measures for this trial was deliberate, however, as people who have experienced stroke are more concerned with being able to complete motor tasks independently and efficiently than with how “normal” a movement appears.

*Power*: The study was slightly underpowered for the WMFT outcome (power = 78%) given higher than anticipated attrition and inability to recruit the target 224 participants. Nonetheless, there were no clinically meaningful differences between groups on the WMFT, so the clinical meaning of the results would be unlikely to change even if more participants were recruited into the study.

*Attrition:*  Attrition threatens internal validity because the sample that completed the study may differ from the sample that began the study. While no observable relationships emerged between attrition during the treatment period and any measured variables, it is possible that data is missing not at random because unmeasured variables (e.g., transportation challenges) may influence attrition. Maintaining participants during the follow-up period was particularly challenging within the treatment groups that had received less contact with a therapist during the treatment period (Self-Gaming and Traditional). Substantial site-dependent and group-dependent attrition during follow-up may adversely influence the validity of the follow-up findings because those who withdrew or were lost to follow-up could have certain unmeasured characteristics (e.g., higher levels of family stress, housing instability, etc.) that could theoretically influence outcomes.^69^ While the use of multiple imputation and intent-to-treat analysis may minimize this bias, they cannot fully eliminate the potential bias arising from nonrandom attrition. Thus, the estimated retention of clinical gains amongst the two groups receiving less therapist contact, as well as the comparative treatment effects in follow-up, should be interpreted cautiously.

*Therapist factors*: Overall, therapists were least skilled at delivering the behavioral elements of the treatment protocol employed within the CI therapy and gaming interventions, as evidenced by occasional retraining needed on these elements (i.e., after incomplete implementation of behavioral techniques was flagged during fidelity monitoring). This is unsurprising given that these treatment elements differ most markedly from current clinical practice and that the majority of therapists who delivered the study interventions had no prior experience delivering these techniques. Thus, the improvements in everyday arm use observed here may slightly underestimate what could occur if therapists had more experience employing these techniques in their clinical practice. In other words, this bias works against the study hypotheses.

# References

1. Gauthier LV, Kane C, Borstad A, et al. Video game rehabilitation for outpatient stroke (VIGoROUS): Protocol for a multi-center comparative effectiveness trial of in-home gamified constraint-induced movement therapy for rehabilitation of chronic upper extremity hemiparesis. *BMC neurology*. 2017;17(1):109.

2. Morris DM, Taub E, Mark VW. Constraint-induced movement therapy: Characterizing the intervention protocol. *Eura Medicophys*. 2006;42(3):257-68.

3. Gauthier LV. CI therapy transfer package. Youtube; 2016.

4. Taub E, Uswatte G, Mark VW, et al. Method for enhancing real-world use of a more affected arm in chronic stroke: Transfer package of constraint-induced movement therapy. *Stroke*. 2013;44(5):1383-1388.

5. Harris JE, Eng JJ, Miller WC, Dawson AS. A self-administered graded repetitive arm supplementary program (GRASP) improves arm function during inpatient stroke rehabilitation: A multi-site randomized controlled trial. *Stroke*. 2009;40(6):2123-2128.

6. Borg G. Borg's perceived exertion and pain scales. Human kinetics; 1998.

7. Pang M, Harris J, Eng J. A community-based group upper extremity exercise program improves motor function and performance of functional activities in chronic stroke: A randomized controlled trial. *Arch Phys Med Rehabil*. 2006;96:145-156.

8. Duncan PW, Sullivan KJ, Behrman AL, et al. Protocol for the locomotor experience applied post-stroke (LEAPS) trial: A randomized controlled trial. *BMC neurology*. 2007;7(1):39.

9. Duncan PW, Sullivan KJ, Behrman AL, et al. Body-weight–supported treadmill rehabilitation after stroke. *N Engl J Med*. 2011;364(21):2026-2036.

10. Veldema J, Jansen P. Resistance training in stroke rehabilitation: Systematic review and meta-analysis. *Clin Rehabil*. 2020:0269215520932964.

11. Karamians R, Proffitt R, Kline D, Gauthier LV. Effectiveness of virtual reality-and gaming-based interventions for upper extremity rehabilitation post-stroke: A meta-analysis. *Arch Phys Med Rehabil*. 2020;101(5):885-896.

12. Borstad A, Crawfis R, Phillips K, et al. In-home delivery of constraint induced movement therapy via virtual reality gaming is safe and feasible: A pilot study. *J Patient Cent Res Rev*. 2017;in press.

13. Maung D, Gauthier LV, Worthen-Chaudhari L, et al. Games for therapy: Defining a grammar and implementation for the recognition of therapeutic gestures. *FDG*. 2013;Crete, Greece.

14. Maung D, Crawfis R, Gauthier LV, et al. Development of recovery rapids-A game for cost effective stroke therapy. *FDG*. 2014.

15. Timmermans AA, Spooren AI, Kingma H, Seelen HA. Influence of task-oriented training content on skilled arm-hand performance in stroke: A systematic review. *Neurorehabil Neural Repair*. 2010;24(9):858-870.

16. Van Peppen RP, Kwakkel G, Wood-Dauphinee S, Hendriks HJ, Van der Wees PJ, Dekker J. The impact of physical therapy on functional outcomes after stroke: What's the evidence? *Clin Rehabil*. 2004;18(8):833-862.

17. Gauthier LV, Taub E, Perkins C, Ortmann M, Mark VW, Uswatte G. Remodeling the brain: Plastic structural brain changes produced by different motor therapies after stroke. *Stroke*. 2008;39(5):1520-5.

18. Langhammer B, Stanghelle JK. Bobath or motor relearning programme? A comparison of two different approaches of physiotherapy in stroke rehabilitation: A randomized controlled study. *Clin Rehabil*. 2000;14(4):361-369.

19. Ma HI, Trombly CA, Robinson-Podolski C. The effect of context on skill acquisition and transfer. *Am J Occup Ther*. 1999;53(2):138-144.

20. Krakauer JW. Motor learning: Its relevance to stroke recovery and neurorehabilitation. *Curr Opin Neurol*. 2006;19(1):84-90.

21. Magill RA. Practice variability and specificity. In: *Motor learning and control: Concepts and applications.* 8th ed. McGraw-Hill; 2007:482.

22. Hanlon RE. Motor learning following unilateral stroke. *Arch Phys Med Rehabil*. 1996;77(8):811-815.

23. Birkenmeier RL, Prager EM, Lang CE. Translating animal doses of task-specific training to people with chronic stroke in 1-hour therapy sessions: A proof-of-concept study. *Neurorehabil Neural Repair*. 2010;24(7):620-635.

24. Orrell AJ, Eves FF, Masters RS. Motor learning of a dynamic balancing task after stroke: Implicit implications for stroke rehabilitation. *Phys Ther*. 2006;86(3):369-380.

25. Cirstea M, Levin MF. Improvement of arm movement patterns and endpoint control depends on type of feedback during practice in stroke survivors. *Neurorehabil Neural Repair*. 2007;21(5):398-411.

26. Rothgangel AS, Braun SM, Beurskens AJ, Seitz RJ, Wade DT. The clinical aspects of mirror therapy in rehabilitation: A systematic review of the literature. *Int J Rehabil Res*. 2011;34(1):1-13.

27. Celnik P, Webster B, Glasser DM, Cohen LG. Effects of action observation on physical training after stroke. *Stroke*. 2008;39(6):1814-1820.

28. Ertelt D, Small S, Solodkin A, et al. Action observation has a positive impact on rehabilitation of motor deficits after stroke. *Neuroimage*. 2007;36 Suppl 2:T164-73.

29. Wolf SL, Winstein CJ, Miller JP, et al. Effect of constraint-induced movement therapy on upper extremity function 3 to 9 months after stroke: The EXCITE randomized clinical trial. *JAMA*. 2006;296(17):2095-2104.

30. Huang H, Wolf SL, He J. Recent developments in biofeedback for neuromotor rehabilitation *J Neuroeng Rehabil*. 2006;3:11.

31. Van de Winckel A, Gauthier L. A revised motor activity log following rasch validation (rasch-based MAL-18) and consensus methods in chronic stroke and multiple sclerosis. *Neurorehabil Neural Repair*. 2019;33(10):787-791.

32. Uswatte G, Taub E, Morris D, Light K, Thompson PA. The motor activity log-28: Assessing daily use of the hemiparetic arm after stroke. *Neurology*. 2006;67(7):1189-94.

33. Hodics TM, Nakatsuka K, Upreti B, Alex A, Smith PS, Pezzullo JC. Wolf motor function test for characterizing moderate to severe hemiparesis in stroke patients. *Arch Phys Med Rehabil*. 2012;93(11):1963-1967.

34. Whitall J, Savin DN,Jr, Harris-Love M, Waller SM. Psychometric properties of a modified wolf motor function test for people with mild and moderate upper-extremity hemiparesis. *Arch Phys Med Rehabil*. 2006;87(5):656-660.

35. Morris DM, Uswatte G, Crago JE, Cook EW,3rd, Taub E. The reliability of the wolf motor function test for assessing upper extremity function after stroke. *Arch Phys Med Rehabil*. 2001;82(6):750-5.

36. Winstein CJ, Wolf SL, Dromerick AW, et al. Effect of a task-oriented rehabilitation program on upper extremity recovery following motor stroke: The ICARE randomized clinical trial. *JAMA*. 2016;315(6):571-581.

37. Chen H, Chen CC, Hsueh I, Huang S, Hsieh C. Test-retest reproducibility and smallest real difference of 5 hand function tests in patients with stroke. *Neurorehabil Neural Repair*. 2009.

38. Cella D, Lai JS, Nowinski CJ, et al. Neuro-QOL: Brief measures of health-related quality of life for clinical research in neurology. *Neurology*. 2012;78(23):1860-1867.

39. Gershon RC, Lai JS, Bode R, et al. Neuro-QOL: Quality of life item banks for adults with neurological disorders: Item development and calibrations based upon clinical and general population testing. *Qual Life Res*. 2012;21(3):475-486.

40. Perez L, Huang J, Jansky L, et al. Using focus groups to inform the neuro-QOL measurement tool: Exploring patient-centered, health-related quality of life concepts across neurological conditions. *J Neurosci Nurs*. 2007;39(6):342-353.

41. Healy BC, Zurawski J, Gonzalez CT, Chitnis T, Weiner HL, Glanz BI. Assessment of computer adaptive testing version of the neuro-QOL for people with multiple sclerosis. *Multiple Sclerosis Journal*. 2019;25(13):1791-1799.

42. Carlozzi NE, Boileau NR, Hahn EA, Barton SK, Cella D, McCormack MK, Ready RE. Responsiveness to Change Over Time: An Examination of the Neuro-QoL Social Function Measures in Persons with Huntington’s Disease. *J Huntingtons Dis*. 2020 Jan 1;9(1):83-97.

43. Hunter J, Mackin E, Callahan A. *Rehabilitation of the hand: Surgery and therapy.* 4th ed ed. Mosby, Incorporated; 1995.

44. Novak CB, Mackinnon SE, Williams JI, Kelly L. Establishment of reliability in the evaluation of hand sensibility. *Plast Reconstr Surg*. 1993;92(2):311-322.

45. Halar EM, Hammond MC, LaCava EC, Camann C, Ward J. Sensory perception threshold measurement: An evaluation of semiobjective testing devices. *Arch Phys Med Rehabil*. 1987;68(8):499-507.

46. Rolke R, Magerl W, Campbell KA, et al. Quantitative sensory testing: A comprehensive protocol for clinical trials. *Eur J Pain*. 2006;10(1):77-88.

47. Yang Z, Rafiei MH, Hall A, et al. A novel methodology for extracting and evaluating therapeutic movements in game-based motion capture rehabilitation systems. *J Med Syst*. 2018;42(12):255.

48. Dong Y, Sharma VK, Chan BP, et al. The montreal cognitive assessment (MoCA) is superior to the mini-mental state examination (MMSE) for the detection of vascular cognitive impairment after acute stroke. *J Neurol Sci*. 2010;299(1):15-18.

49. Luis CA, Keegan AP, Mullan M. Cross validation of the montreal cognitive assessment in community dwelling older adults residing in the southeastern US. *International Journal of Geriatric Psychiatry: A journal of the psychiatry of late life and allied sciences*. 2009;24(2):197-201.

50. Van Buuren S. *Multivariate imputation by chained equations: MICE V1. 0 user's manual.* Leiden: TNO; 2000.

51. Azur MJ, Stuart EA, Frangakis C, Leaf PJ. Multiple imputation by chained equations: What is it and how does it work? *Int J Methods Psychiatr Res*. 2011;20(1):40-49.

52. Shah AD, Bartlett JW, Carpenter J, Nicholas O, Hemingway H. Comparison of random forest and parametric imputation models for imputing missing data using MICE: A CALIBER study. *Am J Epidemiol*. 2014;179(6):764-774.

53. Tang F, Ishwaran H. Random forest missing data algorithms. *Statistical Analysis and Data Mining: The ASA Data Science Journal*. 2017;10(6):363-377.

54. Carranza EJM, Laborte AG. Random forest predictive modeling of mineral prospectivity with small number of prospects and data with missing values in abra (philippines). *Comput Geosci*. 2015;74:60-70.

55. Waljee AK, Mukherjee A, Singal AG, et al. Comparison of imputation methods for missing laboratory data in medicine. *BMJ Open*. 2013;3(8):10.1136/bmjopen-2013-002847.

56. Breiman L. Random forests. *Mach Learning*. 2001;45(1):5-32.

57. Breiman L. Bagging predictors. *Mach Learning*. 1996;24(2):123-140.

58. Hines RO, Carter E. Improved added variable and partial residual plots for the detection of influential observations in generalized linear models. *Journal of the Royal Statistical Society: Series C (Applied Statistics)*. 1993;42(1):3-16.

59. Wolf SL, Thompson PA, Winstein CJ, et al. The EXCITE stroke trial: Comparing early and delayed constraint-induced movement therapy. *Stroke*. 2010;41(10):2309-2315.

60. Appelros P, Stegmayr B, Terént A. Sex differences in stroke epidemiology: A systematic review. *Stroke*. 2009;40(4):1082-1090.

61. Sunderland A, Tinson D, Bradley L, Hewer RL. Arm function after stroke. an evaluation of grip strength as a measure of recovery and a prognostic indicator. *J Neurol Neurosurg Psychiatry*. 1989;52(11):1267-1272.

62. Kozlowski AJ, Cella D, Nitsch KP, Heinemann AW. Evaluating individual change with the quality of life in neurological disorders (neuro-QoL) short forms. *Arch Phys Med Rehabil*. 2016;97(4):650-654. e8.

63. Wu C, Chen C, Tsai W, Lin K, Chou S. A randomized controlled trial of modified constraint-induced movement therapy for elderly stroke survivors: Changes in motor impairment, daily functioning, and quality of life. *Arch Phys Med Rehabil*. 2007;88(3):273-278.

64. Kwakkel G, Veerbeek JM, van Wegen EE, Wolf SL. Constraint-induced movement therapy after stroke. *Lancet Neurol*. 2015;14(2):224-234.

65. Kelly KM, Borstad AL, Kline D, Gauthier LV. Improved quality of life following constraint-induced movement therapy is associated with gains in arm use, but not motor improvement. *Top Stroke Rehabilitation*. 2018;25(7):467-474.

66. van Delden AE, Peper CE, Beek PJ, Kwakkel G. Match and mismatch between objective and subjective improvements in upper limb function after stroke. *Disabil Rehabil*. 2013;35(23):1961-1967.

67. Taub E, Uswatte G, King DK, Morris D, Crago JE, Chatterjee A. A placebo-controlled trial of constraint-induced movement therapy for upper extremity after stroke. *Stroke*. 2006;37(4):1045-9.

68. Wolf SL, Winstein CJ, Miller JP, et al. Retention of upper limb function in stroke survivors who have received constraint-induced movement therapy: The EXCITE randomised trial. *Lancet Neurol*. 2008;7(1):33-40.

69. Britton A, Murray D, Bulstrode C, McPherson K, Denham R. Loss to follow-up: Does it matter? *Lancet*. 1995;345(8963):1511-1512.
